# Heterozygous expression of the Alzheimer’s disease-protective PLCγ2 P522R variant enhances Aβ clearance while preserving synapses

**DOI:** 10.1007/s00018-022-04473-1

**Published:** 2022-07-27

**Authors:** Shiden Solomon, Nirmal Kumar Sampathkumar, Ivo Carre, Mrityunjoy Mondal, George Chennell, Anthony C. Vernon, Marc-David Ruepp, Jacqueline C Mitchell

**Affiliations:** 1 Department of Basic and Clinical Neuroscience, Institute of Psychiatry, Psychology and Neuroscience, King’s College London, London, UK; 2 UK Dementia Research Institute, King’s College London, London, UK; 3 Wohl Cellular Imaging Centre, Institute of Psychiatry, Psychology and Neuroscience, King’s College London, London, UK; 4 Present Address: Alzheimer’s Research UK Oxford Drug Discovery Institute, Centre for Medicines Discovery, University of Oxford, Oxford, UK; 5 MRC Centre for Neurodevelopmental Disorders, King’s College London, London, UK

**Keywords:** Alzheimer’s disease, Microglia, PLCG2, iPSC

## Abstract

**Background:**

A rare coding variant, *P522R*, in the phospholipase C gamma 2 (*PLCG2)* gene has been identified as protective against late-onset Alzheimer’s disease (AD), but the mechanism is unknown. *PLCG2* is exclusively expressed in microglia within the central nervous system, and altered microglial function has been implicated in the progression of AD.

**Methods:**

Healthy control hiPSCs were CRISPR edited to generate cells heterozygous and homozygous for the PLCG2^P522R^ variant. Microglia derived from these hiPSC’s were used to investigate the impact of PLCγ2^P522R^ on disease relevant processes, specifically microglial capacity to take up amyloid beta (Aβ) and synapses. Targeted qPCR assessment was conducted to explore expression changes in core AD linked and microglial genes, and mitochondrial function was assessed using an Agilent Seahorse assay.

**Results:**

Heterozygous expression of the *P522R* variant resulted in increased microglial clearance of Aβ, while preserving synapses. This was associated with the upregulation of a number of genes, including the anti-inflammatory cytokine Il-10, and the synapse-linked CX3CR1, as well as alterations in mitochondrial function, and increased cellular motility. The protective capacity of PLCγ2^P522R^ appeared crucially dependent on (gene) ‘dose’, as cells homozygous for the variant showed reduced synapse preservation, and a differential gene expression profile relative to heterozygous cells.

**Conclusion:**

These findings suggest that PLCγ2^P522R^ may result in increased surveillance by microglia, and prime them towards an anti-inflammatory state, with an increased capacity to respond to increasing energy demands, but highlights the delicate balance of this system, with increasing PLCγ2^P522R^ ‘dose’ resulting in reduced beneficial impacts.

**Graphical abstract:**

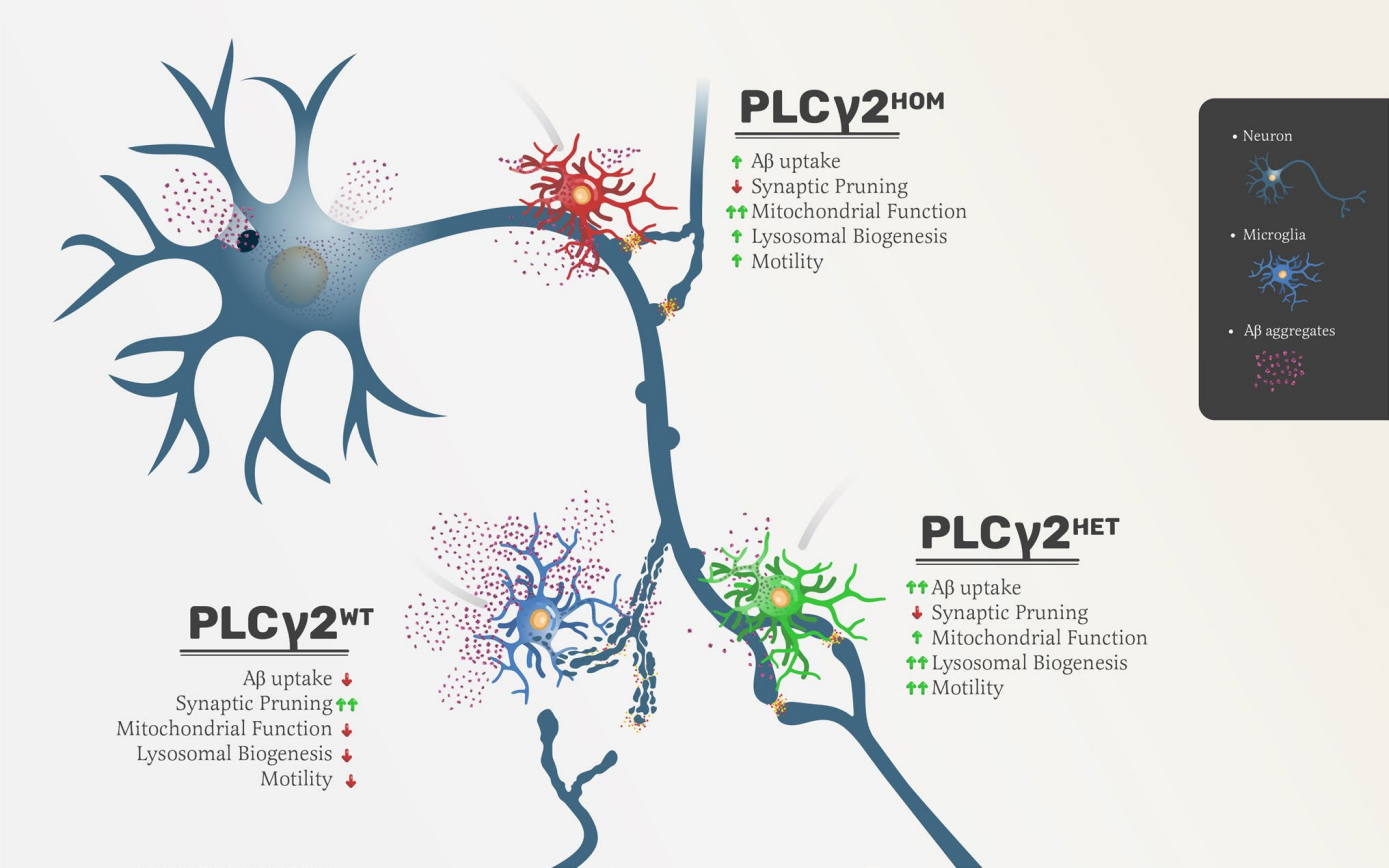

**Supplementary Information:**

The online version contains supplementary material available at 10.1007/s00018-022-04473-1.

## Background

Alzheimer’s disease (AD) is a devastating neurodegenerative disorder that presents a significant and growing global socioeconomic burden. Although significant work has focussed on understanding and modifying the disease process, the development of truly effective therapies has remained elusive. Pathologically, AD is characterised by the formation of extracellular neuritic plaques formed from Aβ peptides, and the intraneuronal accumulation of aberrantly phosphorylated Tau protein, forming neurofibrillary tangles, accompanied by the progressive loss of synapses and neurons [1, 2]. Despite ongoing debate, Aβ accumulation, whether in the form of insoluble plaques, or smaller soluble oligomers does appear to be a primary driver of the disease process, particularly linked to synapse dysfunction [3].

Converging lines of evidence from human genetics, neuroimaging and post-mortem brain tissue studies suggest that microglia are major contributors to molecular and phenotypic changes, including synaptic loss and Aβ clearance, in the AD brain [4]. Microglia constantly survey the brain parenchyma for debris, apoptotic cells, aberrant misfolded proteins, and pathogens [5, 6]. Under normal physiological conditions, microglia also play a pivotal role in maintaining normal brain homeostasis, modulating synaptic strength and plasticity during development [6]. In the presence of pathological stimuli however, microglia undergo a morphological transformation from a highly ramified structure to a more ameboid reactive phenotype [7]. These “reactive” microglia are characterised by rapid proliferation and enhanced secretion of chemokines, cytokines, and immune mediators central to tissue repair and clearance of cellular debris [8]. Crucially, a subset of disease-associated microglia (DAM) has been identified in neurodegenerative disorders such as AD [9], with a profile unique to disease, highlighting the importance of these cells to the disease process.

GWAS studies have identified several novel risk associated coding variants for late-onset AD (LOAD), many of which are associated with microglial and immune function and further link these cells with AD pathogenesis. Among these identified polymorphic genes are rare coding variants in microglial-associated genes such as triggering receptor expressed on myeloid cell-2 (TREM2), ABI family member 3 (ABI3), and phospholipase C gamma 2 (PLCG2) [10]. Specifically, a single nucleotide polymorphism (Pro522Arg; *P522R*) in the PLCG2 gene, which is almost exclusively expressed in microglia within the central nervous system (CNS), has been found to be protective against LOAD [11], further highlighting the importance of these cells in disease.

The protein product of PLCG2 (PLCγ2) is a PIP2 metabolising enzyme that is recruited to the cell membrane upon activation by tyrosine kinase and modulates immune signals impacting cell fate and function, both in the brain and periphery [12, 13]. Recent studies have identified PLCγ2 downstream of TREM2 signalling, modulating different microglial functions such as phagocytosis, lipid metabolism, survival and cytokine release [14, 15]. It also appears to play a role in TLR4 signalling independent of its role in the TREM2 pathway [14], highlighting its role in facilitating inflammatory responses. However, specifically how the *P522R* protective variant modulates microglia activity to protect against AD-related pathologic insult remains unclear.

Recent studies have suggested that the *P522R* variant is a mild hypermorph [15–17], serving to potentiate the enzymatic activity of the protein, thus impacting on its downstream targets. It has been shown that this altered activity appears to enhance macrophage phagocytic activity and enhance cytokine release in response to inflammatory stimulation, and may promote microglial activation [17], priming them to adopt an MHC class II state, that is notably reduced in AD brains [18]. Conversely however, other studies have suggested that in fact phagocytic clearance is impaired, while endocytic clearance is enhanced [15], hence its exact impact on phagocytic vs. endocytic clearance of AD relevant cargoes is still unclear.

In AD, dysregulation of Aβ clearance and microglia-mediated synaptic pruning are two mechanisms by which microglia contribute to disease progression. Therefore a crucial therapeutic strategy in AD, at least from microglia associated function, would be robust clearance of Aβ aggregates while sparing synapses. In this study, we demonstrate that the heterozygous PLCγ2^P522R^ protective variant modulates microglia-mediated Aβ and synaptosome uptake, which may underpin its protective nature in vivo. We show a potential impact of PLCγ2^*P522R*^ on microglia mitochondrial function and ATP production, which may contribute to maintenance of microglia metabolic fitness. In addition, we report enhanced activation status, higher lysosomal activity, and increased motility in PLCγ2 microglia variants. Crucially, we also demonstrate that the protective capacity of the PLCγ2^P522R^ variant is critically linked to its dose (the number of modified alleles), highlighting the need for a better understanding of the specific impact this variant has on microglial function, and how these pathways may be manipulated to improve outcomes in AD.

## Methods

### iPSC line and maintenance

hiPSC line BIONi10-C (Bio Sample ID: SAMEA3158050, ECACC ID:66,540,023) was purchased from EBiSC, which was deposited by Bioneer, and is available for research from European Collection of Authenticated Cell Cultures (ECACC). The PLCγ2 WT locus was authenticated through sequencing (Supplementary Table 1) and the PLCγ2^HET^ and PLCγ2^HOM^ hiPSC lines were generated on the parent BIONi10-C WT background in house using CRISPR editing. hiPSCs were cultured on Geltrex coated (100X) (A1413202, Gibco) plates and maintained at 37 °C and 5% CO_2_ in E8-Flex medium (A2858501, ThermoFisher).

### CRISPR editing

In brief, to insert the P522R variant, BIONi010-C WT hiPSCs were dissociated to single cells using StemPro™ Accutase™ (A1110501, ThermoFisher). Cells were then seeded at 5X10^5^ per well of a 6-well plate and incubated for 3–4 h to allow for attachment. sgRNA complex was prepared by mixing 100 µM stock of trcrRNA (1072532, IDT) and crRNA (sequence—CCTACAGAACTACATTTTGG), targeting exon 17 of the PLCγ2 locus, in equimolar concentration with 196 µL of nuclease free duplex-buffer (IDT) to a final volume of 200 μL and annealing at 95 °C for 5 min. At the same time, 1 µM stock Cas9 RNP was made by adding 1 µL of 62 µM Hi-Fi Cas9 RNP (1081060, IDT) to 61 µL of Opti-MEM™ (31985070, ThermoFisher**)**. 12 µL of sgRNA complex and 12 µL of Cas9 RNP were then mixed in 76 µL of Opti-MEM and incubated at room temperature for 5 min. In parallel, 4 µL of Lipofectamine™ Stem (STEM00008, ThermoFisher) and 96 µL of Opti-MEM were mixed and incubated for 10 min at room temperature to make the transfection mixture. 2 µg of donor plasmid and 500 ng of puromycin selection construct were added to the sgRNA:Cas9 (RNP) complex and transfection mixture to a final volume of 200 µL, and incubated for 10 min. The medium on the plated cells was replaced with 2 mL of pre-warmed Opti-MEM supplemented with RevitaCell™ (100X) (A2644501, ThermoFisher) and transfection mix was added on top in a drop-wise manner before mixing gently through swirling the plate. Cells were left for 4 h in the incubator before medium top-up with E8-flex supplemented with RevitaCell and then being left on overnight. Medium changes were performed until cells reached 70–80% confluency, whereafter single cell selection with 0.25 μM Puromycin (A1113803, ThermoFisher) was done to isolate pure colonies. Single cell colonies were established, expanded and banked. To confirm conversion of 522P to 522R, DNA was extracted using DNeasy Blood & Tissue (69506, QIAGEN) and sent for sequencing (Eurofins Genomics) (sequencing primers are available in supplementary methods Table 1) and positive colonies were identified to be used in downstream assays.

### Generation of iPSC-derived microglia

Microglia-like cells were generated following Haenseler et al. [19]. In brief, embryonic bodies (EBs) were formed through plating and spinning of 3X10^6^ BIONi010-C (BINI-10) (WT, PLCG2^HET^ and PLCG2^HOM^) at 300 g on an AggreWell 800 plate (34,850, StemCell Technologies) in E8-Flex medium supplemented with 50 ng/mL BMP4 (120-05ET, Peprotech), 50 ng/mL VEGF (PHC9394, ThermoFisher) and 20 ng/mL SCF (300-07, PeproTech). 75% medium change per day was performed for 72 h, after which EBs were transferred to a T75 flask and maintained in X-VIVO15 (BE02-060F, Lonza) supplemented with 25 ng/mL IL-3 (PHC0031, ThermoFisher), 100 ng/mL M-CSF (300–25, PeproTech), 2 mM Glutamax (35050061, ThermoFisher) and 0.055 mM β-mercaptoethanol (31350-010, ThermoFisher). Medium was topped up every week and after 4 weeks, emerging precursor cells were collected and differentiated to microglia-like cells for 7 days in microglia medium consisting of 25 mL DMEM/F12 (11330032, ThermoFisher) and 25 mL Neurobasal Plus media (A3582901, ThermoFisher) supplemented with 100 ng/mL M-CSF (300-25, Peprotech), 100 ng/mL IL-34 (200-34, peprotech) and 10 ng/mL GM-CSF (300-03, Peprotech), 2 mM Glutamax (35050061, ThermoFisher), 0.05 5 mM β-mercaptoethanol (31350, Life Technologies), and 0.25 μg/mL Insulin (I9278, Sigma).

### Generation of iPSC-derived cortical neurons

iPSC-derived cortical neuron differentiation was adapted from Fernandopulle et al. [20], with slight modifications. In brief, BIONi010-C (BINI-10) iPSC cell lines (PLCG2^HET^) were dissociated using accutase and seeded as single cells at a density of 5X10^5^ per well of a six-well plate. The cells were left to attach for 4 h. Cells were then transduced in a similar manner to the above protocol of PLCγ2 editing, but in this case using a CRISPR-cas9 RNP to stably integrate a doxycycline inducible NGN2 expression cassette (see supplementary methods—plasmids), into the CLYBL safe harbour site, allowing for the driving of cellular differentiation into cortical neurons. Double selection was performed using Blasticidin S (100 ng/mL) (203350-25MG, Sigma-Aldrich) and mApple markers to identify positive colonies. iPSCs stably expressing NGN2 were then induced in DMEM/F12 with HEPES (11330032, ThermoFisher) supplemented with; N2 (1X) (17502048, ThermoFisher), NEAA (1X) (11140050, ThermoFisher), Glutamax (1X) (35050038, ThermoFisher) and doxycycline (2 μg/mL) (D9891, Sigma). 72 h post-induction, the cells were transferred to PDL (A3890401, ThermoFisher) and Laminin (1:100 dilution in PBS) (23017015, ThermoFisher) coated plates and fed with Neurobasal Plus media (A3582901, ThermoFisher) supplemented with B27 (50X) (17504044, ThermoFisher), 10 μg/mL BDNF (450–02, PeproTech), 10 μg/mL NT-3 (450–03, PeproTech) and 1 μg/mL Laminin for up to 12 days.

### BV2 culture

BV2 cells (a gift from Prof. Frei, UZH) were cultured (140,000/well of a 12-well plate, 14,000 per EBD plate, 7,000/well of a 96-well plate) and maintained at 37 °C, 95% O_2_ and 5% CO_2_ in Dulbecco’s modified Eagle’s medium (31,885–023, Gibco) supplemented with 10% (v/v) foetal calf serum (A4768801, ThermoFisher) and 1% pen/strep (15,140,122, ThermoFisher).

### Overexpression of PLCG2 in BV2 cell line

The over-expression construct PLCγ2^P522R^ point mutation was inserted into the human PLCγ2^WT^ plasmid (RC200442, Origene) using Q5® site-directed mutagenesis kit (E0554S, NEB) according to manufacturer’s protocol. The mutagenesis primers used are listed in supplementary Table 1. For over-expression experiments, in brief BV2 cells were seeded onto either a 12-well plate or 8-well chamber slide (IB-80827, iBIDI) at a density of 140,000 and 21,000 cells per well, respectively, and allowed to settle for 24 h. 2 h prior to transfection a complete media change was done with fresh DMEM without pen/strep. For transfection, 50 ηg/100 μL of cDNAs (Control, PLCG2^WT^ and PLCG2^P522R^ plasmid) were mixed with 25 μL/100 μL Opti-MEM^®^ and 0.27 μL/100 μL of Lipofectamine™ 2000 (ThermoFisher) and incubated for 30 min. Afterwards, the media on the cells were replaced with transfection mix and incubated for 24 h. The media was then exchanged for fresh DMEM, and cells were incubated for a further 24 h before downstream assays, including uptake and metabolic profiling.

### Protein extraction and immunoblotting

Protein was extracted from cultured BV2 cells or 7-day mature iPSC-derived microglia by lysis for 30 min at 4 °C in ice-cold RIPA buffer (150 mM NaCl, 10 mM Tris, pH 7.2, 0.1% SDS, 1% Triton X-100, 1% deoxycholate, 5 mM EDTA), supplemented with protease (78430, ThermoFisher) and phosphatase inhibitor (524625-1SET, MERCK) cocktails (ThermoFisher). The RIPA soluble fraction was obtained by centrifugation of the samples at 10,000×*g* for 10 min. Protein concentration was determined using the Pierce BCA Protein Assay Kit (Thermo Fisher Scientific). Samples were mixed with loading buffer (928-40004, Li-Cor) before boiling at 95 °C. SDS-PAGE separation (15–20 μg protein per sample) was performed on NuPAGE 4–12% gradient Bis–Tris gels (Thermo Fisher) and proteins were transferred to nitrocellulose membranes (IB23002, ThermoFisher) using an iBLOT2 following manufacturers protocol. Membranes were blocked for 1 h in 3% BSA in TBST (0.1% tween) before incubation with primary antibodies (supplementary Table 2) in 3% BSA in TBST overnight at 4 ^0^C. Membranes were washed 3 times 5 min and incubated with secondary antibodies (supplementary Table 2) for 1 h at room temperature before further washes using TBST. Membranes were analysed using a LI-COR Odyssey CLx system and protein levels were quantified using Image Studio Lite (LI-COR Biosciences), normalised to their corresponding β-Actin levels. Statistical significance was assessed using one-way ANOVA.

### Phagocytosis assay

For the phagocytosis assay 5 × 10^4^ iMG progenitors were seeded onto 8-well chamber slide (IB-80827, iBIDI) coated with PDL and matured for 7 days with 50% medium change every other day. Uptake assays were then performed by exchanging 100% media to fresh media supplemented with either 250 ηM Aβ_1–42_ HyLite Fluor 647 (AS-60493, AnaSpec), mouse brain purified ^td^Tomato-tagged synaptosomes generated in house, FITC-Dextran 4kD (46944, Sigma), FITC-Dextran 150 kD (46946, Sigma), Zymosan beads-568 (Z23374, ThermoFisher) and 75 nM LysoTracker DND-99 (L7528, ThermoFisher) for 100 min. After elapsed time, iMG were washed with PBS and fixed with 4% paraformaldehyde for 30 min. Fixed iMG were subsequently stained with SNL (FL-1301-2, Vector) to visualise the membrane and with 2 µM Hoechst (62249, ThermoFisher) nuclear stain. Images were acquired using Nikon A1R inverted confocal microscope (Nikon) running the NIS elements software using 60 × oil immersion lens. Acquired images were analysed using FIJI and IMARIS, thresholding to WT/Control, marking ROI and quantified either as % Cell Area or uptake (cargo) volume per total cell volume. Statistical significance was assessed using a Kruskal–Wallis test.

### Immunochemiluminescence assay (meso-scale discovery)

To measure the residual level of Aβ in the co-culture assay, supernatant was collected from co-culture of 21-day mature iPSC-derived cortical neurons and 7-day mature microglia and centrifuged at 1000G to remove any cellular debris. In brief, immunochemiluminescence assay was performed by adding 150 μL of Diluent 35 (MSD) to each well of a 96-well multi-spot MSD plate (N45197A-1, MSD) and incubated at room temperature for 1 h, on a plate shaker. Subsequently, the plate was washed 3 times with Tris Wash Buffer (MSD). After washing, 25 μL of Aβ detection antibody (6E10, MSD) and 25 μL of prepared samples (cellular supernatant) was added to each well. The plate was incubated at room temperature for 3 h, on a plate shaker, prior to washing 4 times with Tris Wash Buffer. 150 μL of 2X Read Buffer (MSD) was added to each well. Samples were analysed on the MSD plate reader using default detection criteria. The MSD read outputs, indicative of Aβ level, were normalised to their corresponding total protein concentration and were analysed using GraphPad. Statistical significance was assessed using a Kruskal–Wallis test.

### RT-qPCR analysis

RNA was extracted from iPSC-derived microglia (iMG) and/or BV2 using Direct-zol RNA MiniPrep Kit (Zymo research) according to the manufacturer’s protocol. For RT-qPCR analysis, RNA was reverse transcribed into cDNA using iScript cDNA synthesis kit (BIO-RAD) according to the manufacturer’s protocol. Target specific PCR primers for mouse and human (Supplementary Table 3) were obtained from IDT. For qRT-PCR analysis Takyon Rox SYBR MasterMix dTTP blue (Eurogentec) was used. For relative expression analysis, the ΔΔCt comparative method was used to compare TBP-normalised expression level of the target mRNA. Data were normalised either to control (BV2) or WT groups (iMG) and are shown as median ± SD. Statistical significance for each gene was assessed using a Kruskal–Wallis test.

### Cellular respiration assay

To measure oxygen consumption rates (OCR) and extracellular acidification rates (ECAR) in real time, iMG were seeded and matured for 7 days on a Seahorse 96-well cell-culture microplate (PerkinElmer) at a density of 25,000 cells per well. Mature iMG’s mitochondrial respiration was then analysed on the seahorse XFe96 Analyser (Agilent Technologies) using a Mitostress test kit (Agilent Technologies) according to manufactures protocol.

For BV2 cells, to measure OCR and ECAR, cells were seeded on a seahorse cell-culture plate at a density of 27,000 cells per well and transfected, as described, with cDNA (Control, WT and PLCG2-P522R) constructs. 24 h post-transfection, real-time mitochondrial respiration was measured using a Mitochondrial stress test kit (Agilent Technologies) on the seahorse XFe96 Analyser. All data were analysed using Wave v2.4.0.6 (Agilent technologies) and Graph pad. Statistica significance was assessed using a Kruskal–Wallis test.

### Cell tracking/motility assay

To measure microglia motility, speed, and distance, iMG were plated on a glass bottom Ultra cell carrier 96-well plate (6055302, PerkinElmer), precoated with PDL, and matured for 7 days as previously described. Mature iMG were stained with nuclear mask blue (ThermoFisher) and cell mask orange (ThermoFisher) for 30 min. Cells were then washed with PBS twice and 100 μL of pre-warmed microglia media made in FluoroBrite (A1896701, Gibco) was added. iMGs were imaged for 2 h on a high-throughput imaging OperaPhenix (PerkinElmer) with both temp and CO_2_ maintained at 37 °C and 5%, respectively. Image analysis was done using Harmony cell tracking software. Both tracked nuclear speed and cytoplasmic speed were exported and analysed using Graphpad.

### Calcium imaging

To monitor Ca^2+^ levels in iMG, microglia progenitors were seeded on a glass bottom cell carrier Ultra 96-well plate and glass bottom 8-well chamber ibidi slides, precoated with PDL. iMG were matured for 7 days following the described protocol. To treat cells, mature iMG were washed with PBS once and 100 μL FloBright microglia media supplemented with cell permeable 2 μM Fura Red™, AM, cell permeant (F3021, ThermoFisher) was added to the 96-well plate and 300 μL was added to the iBidi plates and incubated for 30 min. Cells were then washed with PBS and FloBright microglia media devoid of Fura Red was added. Images were taken on a high-throughput microscope OperaPhenix (PerkinElmer) and Nikon spinning disc (Nikon), with analysis being performed using Harmony software and ImageJ. Background was subtracted from each frame and ratio-metric value of bound to unbound Ca^2+^ was extracted and analysed in GraphPad.

## Results

### PLCγ2 P522R modulates microglia-mediated uptake of Aβ and synaptosomes

**Fig. 1:**
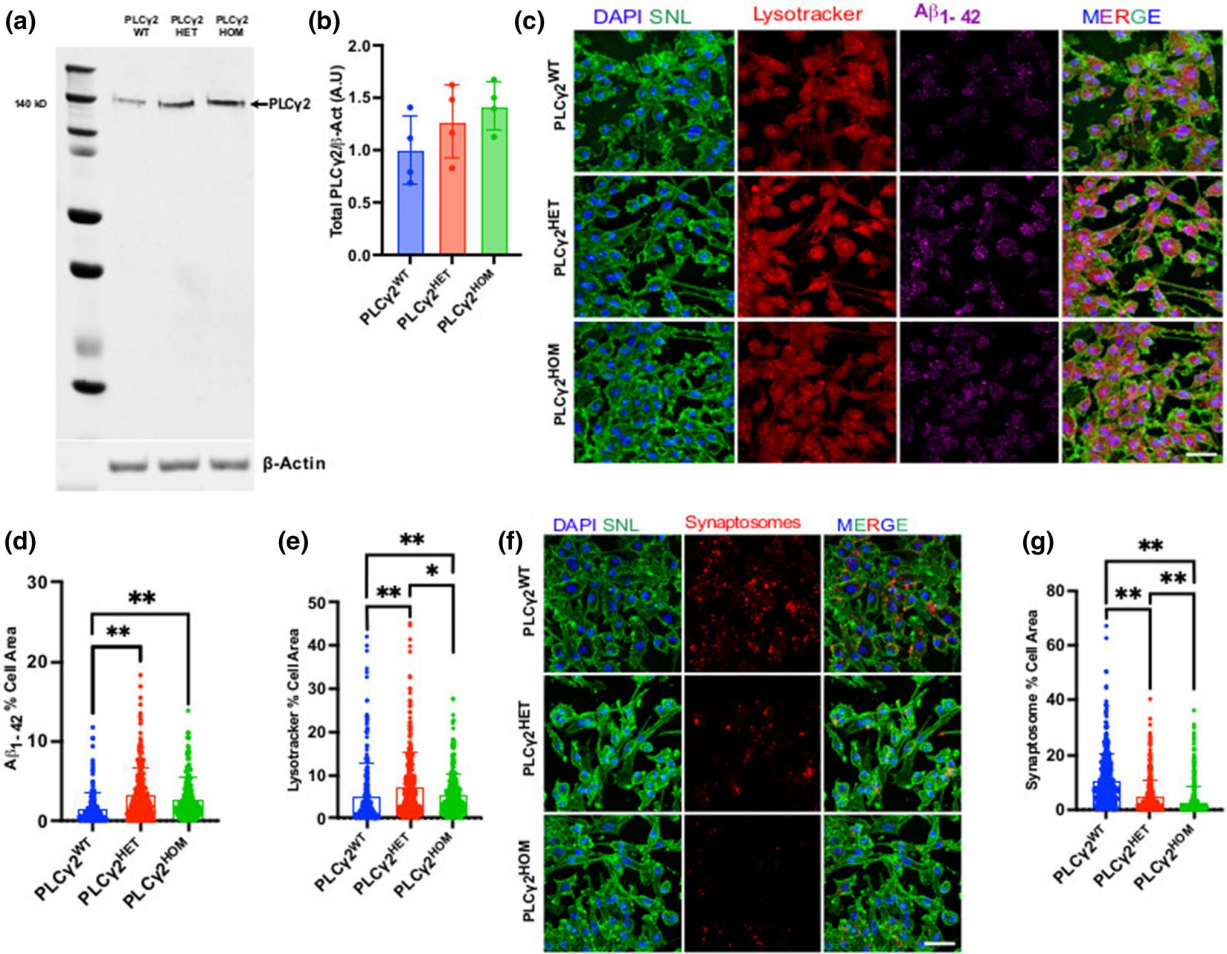
PLCγ2^P522R^ enhances Aβ uptake while reducing synaptosome uptake. **a, b** Western blot analysis of total PLCγ2 protein level in PLCγ2 wild type (PLCγ2^WT^), heterozygous (PLCγ2^HET^), and homozygous (PLCγ2^HOM^) hiPSC-derived microglia cells. Expression levels were normalised to β-actin. Data are presented as mean ± SD and analysed using one-way ANOVA with the Tukey multiple comparisons test. **c–e** Fluorescent confocal analysis of Aβ_1–42_ HyLite Fluor 647 uptake (magenta) and lysotracker levels (red) in PLCγ2^WT^, PLCγ2^HET^ and PLCγ2^HOM^ hiPSC-derived microglia cells. Internalised Aβ (**d**) and lysosome (**e**) levels are expressed as a proportion of total cell area, detected using SNL stain (green). **f–g** Fluorescent confocal analysis of tdTomato-synaptosome uptake (red) in PLCγ2^WT^, PLCγ2^HET^ and PLCγ2^HOM^ hiPSC-derived microglia cells. Internalised synaptosome levels are expressed as a proportion of total cell area, detected using SNL stain (green). Data are presented as mean ± SD and unless otherwise described was analysed using the Kruskal–Wallis with Dunns multiple comparisons test (*p < 0.05, **p < 0.01, *n* = 3–9, scale bar 50 μM)

To investigate the protective role of PLCγ2^P522R^ in human microglia-like cells, we generated isogenic hiPSCs harbouring the *P522R* polymorph in hetero- and homozygosity using CRISPR-mediated gene editing, and differentiated them into microglia-like cells using a previously described protocol [21]. Upon differentiation, PLCγ2^WT^, PLCγ2^HET^ and PLCγ2^HOM^ did not display any obvious phenotypic or karyotypic defects and robustly expressed microglia/macrophage-associated markers including TMEM119 and IBA1 (S. Figure 1). Western blot analysis of PLCγ2 protein levels in the cells detected no significant differences between the three lines (Fig. 1A, B).

**Fig. 2:**
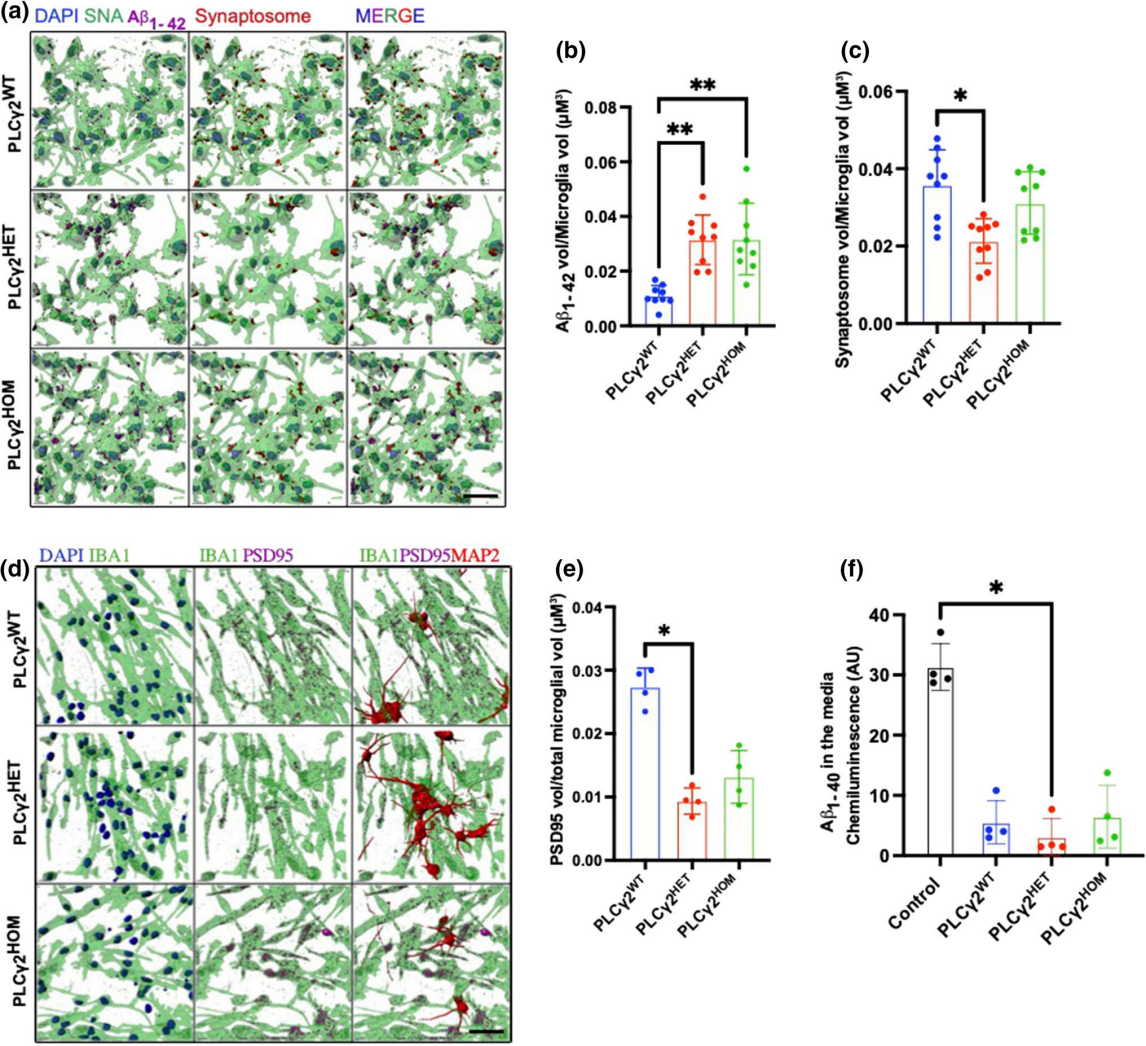
PLCγ2^P522R^ reduces synaptic pruning in hiPSC-derived cortical neuron and microglia co-culture. **a–c** IMARIS 3D reconstruction analysis of Aβ_1–42_ HyLite Fluor 647 (magenta) and tdTomato-synaptosome (red) uptake in PLCγ2^WT^, PLCγ2^HET^ and PLCγ2^HOM^ hiPSC-derived microglia following incubation with both cargoes concurrently. Data are expressed as a proportion of total cell volume, calculated using SNA (*Sambucus nigra* Lectin) staining (green). **d–e** IMARIS 3D reconstruction analysis of synapse uptake by PLCγ2^WT^, PLCγ2^HET^ and PLCγ2^HOM^ hiPSC-derived microglia in neuronal-microglial co-culture. Synaptic elements within microglia were detected using PSD95 immunofluorescence (magenta), microglia cells were identified using Iba1 (green), and iPSC-derived cortical neurons were visualised using MAP2 (red). Synaptic uptake was expressed as a proportion of total microglial cell area (**e**). **f,** Electrochemiluminescence quantification of Aβ_1–40_ levels in media harvested from microglia-neuronal co-culture plates. Aβ_1–40_ release into the media from cortical neurons was also assessed in the absence of microglia (control). Data are expressed as chemiluminescent signal intensity. All data are presented as mean ± SD and were analysed using the Kruskal–Wallis with Dunns multiple comparisons test (*p < 0.05, **p < 0.01, *n* = 3–4, scale bar 50 μM)

To understand the role of PLCγ2^P522R^ in two key aspects of neuroAD pathology, we examined whether it affects microglial-mediated Aβ clearance and synaptic pruning. To this end, we incubated PLCγ2^WT^, PLCγ2^HET^ and PLCγ2^HOM^ iPSC-derived microglia with fluorescently tagged Aβ_1-42_ or ^tD^tomato tagged synaptosomes purified from mouse brain. Microglia expressing the PLCγ2^P522R^ variant demonstrated a robust increase in Aβ uptake compared to the control (Fig. 1C, D), irrespective of heterozygosity. We also saw an increase in lysotracker levels, indicating increased acidic endolysosomal vesicles, in PLCγ2 variants compared to the PLCγ2^WT^, with PLCγ2^HET^ having slightly higher levels than PLCγ2^HOM^ (Fig. 1C, E). By contrast, synaptosome uptake was significantly reduced in PLCγ2^P522R^ variants with a dose-dependent reduction between PLCγ2^HET^ and PLCγ2^HOM^ variant cells (Fig. 1F, G). To further establish whether these findings were associated with the PLCγ2^P522R^ variant, and not a consequence of off-target effects, we transfected BV2 cells, with either a Control, PLCγ2^WT^ or PLCγ2^P522R^ construct, followed by incubation with Aβ_1-42_ peptides and synaptosomes. PLCγ2 expression per se resulted in a robust increase in Aβ uptake compared to the control (S. Figure 2) and a non-significant mild enhancement (*p* = 0.3385) was seen in PLCγ2^P522R^ expressing cells. Furthermore, Aβ accumulation was predominately observed in subcellular compartments, such as lysosomes, while synaptosomes were internalised by the control, but appeared predominantly bound to the cellular membrane in *P225R* mutants (S. Figure 2). In these cells, PLCγ2 is effectively over-expressed; hence, these data primarily demonstrates an impact of increased PLCγ2 expression per se on Aβ and synaptosome uptake. Since the P522R variant appears to be a mild hypermorph [16], it is likely that mild changes induced by the presence of this variant are masked by the larger impact of PLCγ2 over-expression. However, the differential localisation of synaptosomes between PLCγ2^WT^ and PLCγ2^P522R^ over-expression suggests that the impact of the variant on synaptosome uptake may be linked not only to altered protein levels, but also to a possible functional change.

**Fig. 3:**
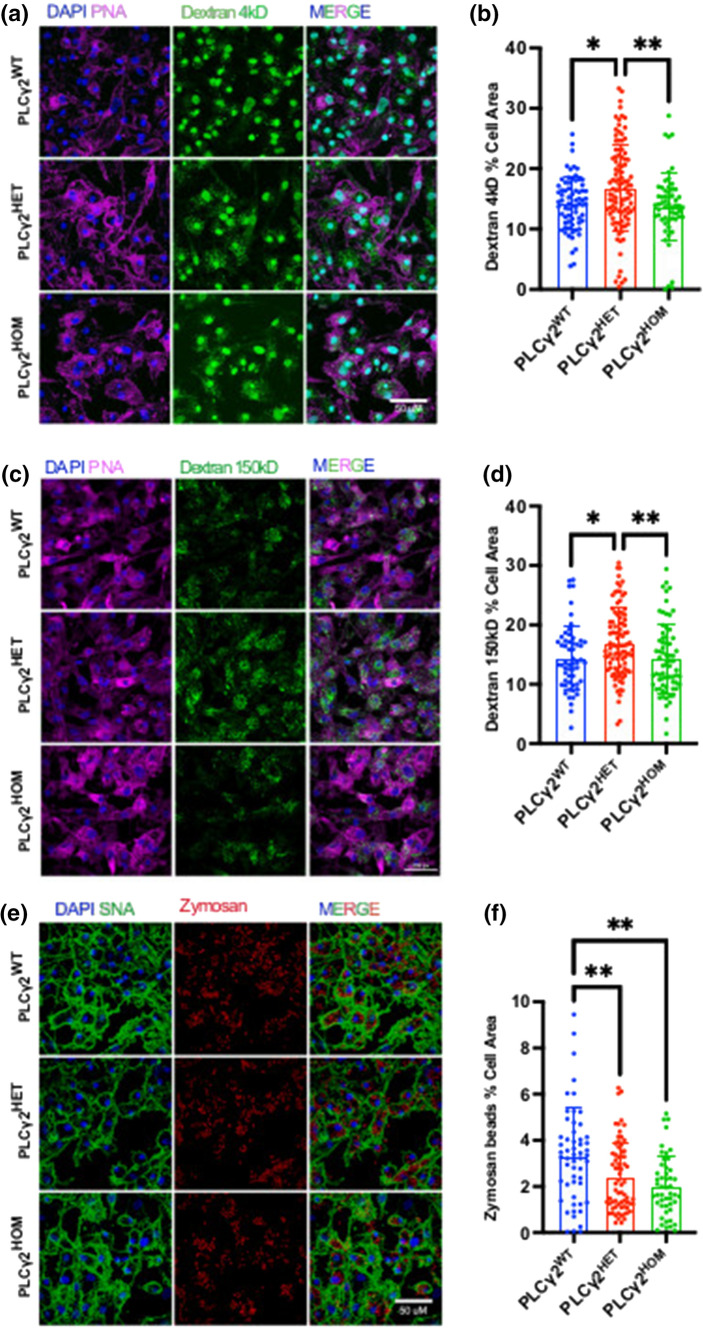
PLCγ2^P522R^ modulation of cargo uptake is size dependent. **a–d** Fluorescent confocal analysis of 4 kDa (**a**–**b**) or 150 kDa (**c**–**d**) FITC-dextran (green) uptake by PLCγ2^WT^, PLCγ2^HET^ and PLCγ2^HOM^ hiPSC-derived microglia. Internalised dextran is expressed as a proportion of total cell area, detected using PNA (Peanut Agglutinin) stain (magenta). **e–f** Fluorescent confocal analysis of Alexa Fluor™ 594-zymosan A Bioparticles™ (red) uptake by PLCγ2^WT^, PLCγ2^HET^ and PLCγ2^HOM^ hiPSC-derived microglia. Internalised zymosan particles are expressed as a proportion of total cell area, detected using SNA stain (green). All data are presented as mean ± SD and were analysed using the Kruskal–Wallis with Dunns multiple comparisons test (*p < 0.05, **p < 0.01, *n* = 3, scale bar 50 μM)

In addition, BV2 cells generally display a small round body with no obvious extensions, similar to the amoeboid-like state reported in vivo [22]; however, when transfected with PLCγ2 construct these cells displayed a bigger, elongated phenotype with several cytoplasmic extensions, possibly indicating a more ramified state (S. Figure 3). Microglial ramifications are typically indicative of resting state microglia, which play an active role in the surveillance and monitoring of the brain parenchyma, with studies showing that insults such as injury result in reduced ramification [23]. It is therefore possible that PLCγ2 may play a role in the regulation of microglial surveillance. A previous study has suggested that the *P522R* variant may promote microglial activation [17], hence indicating a role for this protein in microglial activation state.

It has been suggested that Aβ may facilitate synaptic pruning by microglia by acting as a tag for targeting synapses [24]. To investigate whether the hiPSC microglia variants would specifically target Aβ peptides for clearance or indiscriminately target Aβ and synaptosomes, we incubated mature hiPSC-derived microglia with Aβ and synaptosomes concurrently. 3D Image analysis showed increased Aβ uptake by PLCγ2 microglia variants compared to controls, with no clear significant difference between the PLCγ2^HET^ and PLCγ2^HOM^ lines (Fig. 2A, B). Synaptosome uptake on the other hand was lower in the PLCγ2^HET^ microglia compared to both PLCγ2^WT^ and PLCγ2^HOM^ cells (Fig. 2A, C), suggesting that the ‘dose level’ of the *P522R* variant plays a key role in its altered function.

### PLCγ2^P522R^ affects synaptic pruning in hiPSC-derived cortical neuron and microglia co-culture

The monoculture assays strongly suggest that the PLCγ2^P522R^ protective variant modulates the uptake of Aβ and synaptosomes in an opposing manner. However, this work was conducted in monocultures, which lack the complex neuronal architecture and multicellular networks seen in vivo. Hence, we replicated our initial experiments in a neuronal-microglial cell co-culture system. PLCγ2^WT^, PLCγ2^HET^ or PLCγ2^HOM^ microglia progenitor cells were added to 2-week-old iPSC NGN2-derived cortical neurons, bearing the PLCγ2^HET^ polymorph, and kept for a further 7 days in co-culture allowing the microglia to reach maturation. As expected, microglia were actively involved in synaptic pruning, as measured by PSD95 engulfment, in all conditions tested (Fig. 2D–F). However, the PLCγ2^HET^ variant demonstrated significantly reduced uptake of PSD95 compared to the PLCγ2^WT^ variant (Fig. 2D, E). Interestingly, no significant difference was observed with the PLCγ2^HOM^ microglia. These data highlight our finding that the maximal beneficial impact on synapse pruning is seen in cells heterozygous for the variant. The disparity between our mono- and co-culture findings further suggests that additional mechanisms involved in synaptic pruning beyond phagocytic uptake per se may be subtly impacted by the presence of PLCγ2^P522R^. We also collected supernatant from the co-culture assay to probe for Aβ_1–40_ levels (released endogenously by the iPSC-derived neurons) using an MSD plate reader. While all conditions containing microglia recorded reduced Aβ level compared to neuronal cells in the absence of microglia, only cultures containing microglia heterozygous for the PLCγ2^P522R^ variant reached significance (*p* = 0.036), indicative of increased Aβ uptake by these cells (Fig. 2F). These results collectively establish that heterozygous PLCγ2^P522R^ expression enhances the clearance and modulation of Aβ peptides while sparing synapses in a co-culture setup, providing a possible mechanism for AD protection.

### PLCγ2 P522R differential selectivity is driven by cargo size

PLCγ2^P522R^ has previously been reported to influence selective cargo uptake, suggesting a shift towards endocytic pathways [15]. Given the differential impact of heterozygous and homozygous PLCγ2^P522R^ on Aβ and synaptosome uptake, we sought to further investigate whether similar cargo selectivity differences were observed in our microglial cell model. We assessed hiPSC-derived microglial uptake of Zymosan beads of approximately 3 μm diameter and two dextran glucans of 150 kD and 4 kD. Consistent with our findings for Aβ, PLCγ2^HET^ microglia had a significantly higher uptake of Dextran^4kD^ and Dextran^150kD^ compared to the PLCγ2^WT^ and PLCγ2^HOM^ variants (Fig. 3A–D). Likewise, both PLCγ2^HET^ and PLCγ2^HOM^ cells showed a reduced uptake of zymosan particles compared to PLCγ2^WT^ microglia (Fig. 3E, F), consistent with the previously reported shift towards the endocytic uptake of smaller cargoes.

**Fig. 4:**
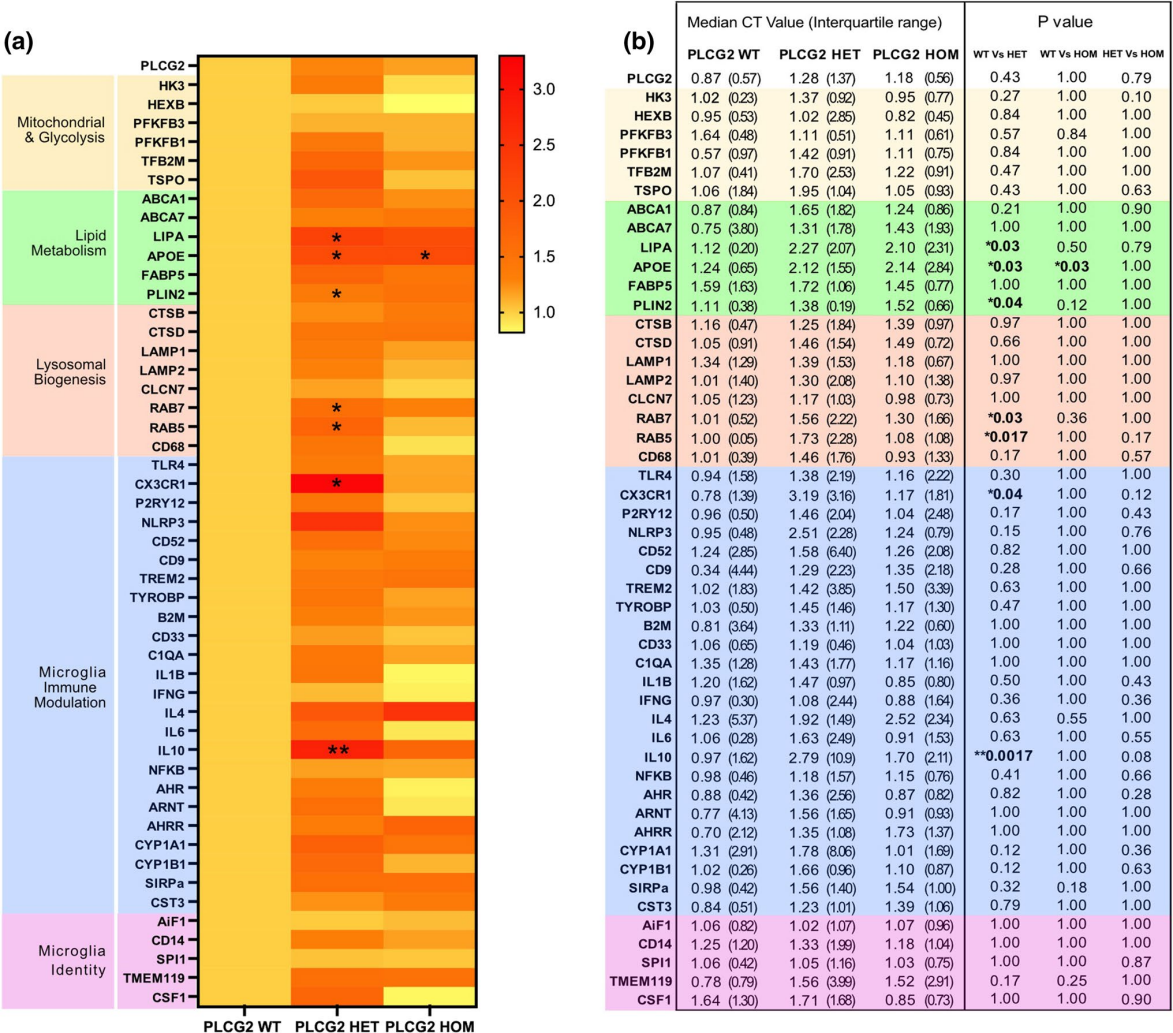
PLCγ2^P522R^ modulates expression of a number of microglial genes. **a** Heatmap demonstrating expression fold-changes detected vis qPCR analysis in selected genes in PLCγ2^WT^, PLCγ2^HET^ and PLCγ2^HOM^ hiPSC-derived microglia. **b,** Table showing the qPCR derived median CT value and interquartile range indicating the relative expression of selected genes in PLCγ2^WT^, PLCγ2^HET^ and PLCγ2.^HOM^ hiPSC-derived microglia, and the respective *p* values obtained following Kruskal–Wallis with Dunns multiple comparisons test analysis for each gene (*p < 0.05, **p < 0.01, *n* = 7)

Similar studies in BV2 cells over expressing either WT or *P522R* PLCγ2 showed that over-expression of the protein, irrespective of the variant, resulted in a marked increase in zymosan uptake, and reduced uptake of both the dextran substrates, supporting the notion that PLCγ2 plays a key role in the regulation of microglial uptake size-selectivity. For both zymosan and 4 kDa Dextran, the *P522R* variant displayed a trend towards reduced effects compared to WT over-expression, in contrast, the *P522R* variant significantly enhanced uptake of Dextran^150kD^ (S. Figure 4).

### PLCγ2 P522R modulates multiple functions of hiPSC-derived microglia

To begin to unpick the cellular pathways that may play a role in PLCγ2^P522R^ altered uptake of AD relevant cargoes, we investigated the effect of the variant on the expression profiles of a number of functionally relevant microglial genes. We focussed on pathways previously identified as potentially playing a role in AD progression, as well as core genes involved in modulating microglial immune function and functional status.

We harvested 7-day differentiated hiPSC-derived microglia in the absence of additional stimulus (‘resting’) and probed the expression profile of a selection of different genes (Fig. 4). We saw significant increases in the expression of a small number of genes associated with lipid metabolism (LIPA; *p* = 0.0374, APOE; *p* = 0.0331, PLIN2; *p* = 0.0422), endosome/phagosome maturation (RAB5; *p* = 0.0351, RAB7; *p* = 0.0174) and cytokines/chemokines (IL-10; *p* = 0.0043, CX3CR1; *p* = 0.0475) in PLCγ2^HET^ cells compared to PLCγ2^WT^ cells. Only one of these genes, APOE, was found to be significantly increased in PLCγ2^HOM^ cells (*p* = 0.0374). A small number of additional genes also showed a trend towards increased expression in PLCγ2^HET^ cells, although these did not reach significance (CD68; *p* = 0.1656, P2RY12; *p* = 0.1739, NLRP3; *p* = 0.1498, CYP1A1; *p* = 0.1209, CYP1B1; *p* = 0.1219, TMEM119; *p* = 0.1739). Although we did not see any significant differences between PLCγ2^HET^ and PLCγ2^HOM^ cells, there was a trend towards significant differences in RAB5 (*p* = 0.1737), CX3CR1 (*p* = 0.1221) and Il-10 (*p* = 0.0753), highlighting the potential importance of these genes in the functional impact of the PLCγ2^P522r^ variant. These findings suggest that PLCγ2^HET^ cells display a subtly different expression profile to both control and PLCγ2^HOM^ cells even in a resting state, that may underpin the protective effects of the *P522R* variant seen in our functional assays and in AD.

### PLCγ2 P522R enhances mitochondrial oxidative phosphorylation in hiPSC-derived microglia

**Fig. 5:**
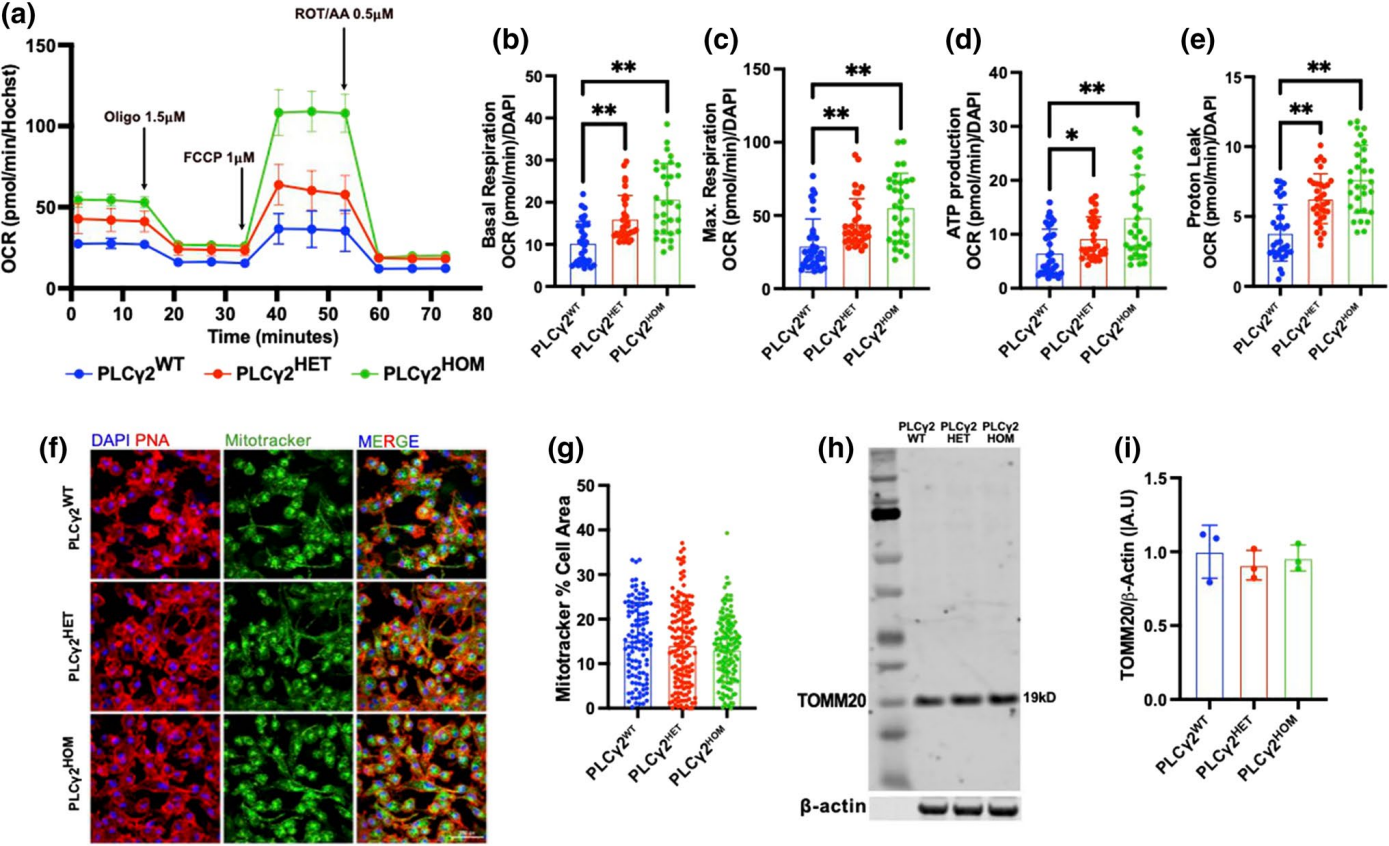
PLCγ2^P522R^ modulates mitochondrial activity but not abundance. **a–e** Real time oxygen consumption rate (OCR) at baseline and in response to oligomycin (ATP-synthase inhibitor), FCCP (mitochondrial membrane uncoupler) and rotenone/antimycin-A (Complex I and III inhibitors) in PLCγ2^WT^, PLCγ2^HET^ and PLCγ2^HOM^ hiPSC-derived microglia. Calculated basal respiration (**b**), maximal respiration (**c**) ATP production (**d**) and proton leak (**e**) are reported. **f–g** Fluorescent confocal analysis of mitochondrial abundance in PLCγ2^WT^, PLCγ2^HET^ and PLCγ2^HOM^ hiPSC-derived microglia. Mitochondria were visualised using MitoTracker Green^FM^ (green), and expressed as a proportion of total cell area, detected using PNA stain. Data are presented as mean ± SD and analysed using the Kruskal–Wallis with Dunns multiple comparisons test. **h–i** Western blot analysis of mitochondrial protein TOMM20 in PLCγ2^WT^, PLCγ2^HET^ and PLCγ2^HOM^ hiPSC-derived microglia. Expression levels were normalised to β-actin. Data are presented as mean ± SD and analysed using one-way ANOVA with the Tukey multiple comparisons test (*p < 0.05, **p < 0.01, *n* = 3–4. Scale bar 50 μM)

The bioenergetic profile of microglia is directly linked to their functional state. Given that the PLCγ2^P522R^ variant impacts on metabolic and mitochondrial biogenesis pathways and shows increased Aβ uptake, an energy-demanding process, we speculated that it may impact directly on mitochondrial function. To investigate the role of PLCγ2 on mitochondrial respiration we employed an Agilent Seahorse assay to look at the mitochondrial oxygen consumption rate (OCR), a measure of mitochondrial function. Under a mitochondrial stress test paradigm, PLCγ2^HET^ and PLCγ2^HOM^ cells showed a dose-dependent increase in basal and maximal respiration compared to PLCγ2^WT^ cells (Fig. 5A–C). ATP production was also increased (Fig. 5D). Proton leak (the migration of protons across the mitochondrial membrane independent of ATP-synthase) was dose dependently higher in the PLCγ2^P522R^ cells compared to the PLCγ2^WT^ variant (Fig. 5E). It has been suggested that increased proton leak is directly related to the prevention of oxidative stress, the inhibition of fatty acid induced mitochondrial damage and the control of oxidative phosphorylation [25], potentially protecting the cell from excessive damage. Hence, our findings suggest superior mitochondrial performance in PLCγ2^P522R^ variants.

To check whether increased mitochondrial function was simply a result of increased mitochondrial number, we incubated matured microglia with mitotracker. Confocal imaging revealed a comparable level of mitotracker in all PLCγ2 variant cells, thus mitochondrial number is unlikely to be affected by the presence of the *P522R* variant (Fig. 5F, G). We also probed for TOMM20 (Translocase Of Outer Mitochondrial Membrane 20); part of the mitochondrial receptor complex responsible for the recognition and translocation of cytosolically synthesised mitochondrial preproteins. Western blot analysis revealed no difference in TOMM20 protein expression between PLCγ2^P522R^ and PLCγ2^WT^ cells (Fig. 5H, I). These data suggest that altered mitochondrial function was solely responsible for enhanced OCR and ATP production as opposed to changes in mitochondrial number.

To validate our hiPSC-derived microglia findings, we again transfected BV2 cells with the WT or *P522R* variant PLCγ2, or control constructs and monitored OCR in real time using the seahorse extracellular flux analyser. Consistent with our hiPSC-derived cells, BV2 cells overexpressing PLCγ2^P522R^ and PLCγ2^WT^ had significantly higher levels of basal respiration, maximal respiration and ATP production compared to those transfected with the control construct (SFig 5). Interestingly, while maximal respiration in cells over expressing PLCγ2^P522R^ was significantly lower than cells over expressing the WT protein, the ATP production was comparable, indicating an optimal level of mitochondrial function in PLCγ2^P522R^ cells that efficiently met the ATP demand (S. Figure 5C, D). Likewise, BV2 cells with PLCγ2 over-expression showed a higher level of proton leak compared to controls, with no clear difference between PLCγ2^WT^ and PLCγ2^P522R^ (S. Figure 5E). In conclusion, we identified a novel role of PLCγ2 on mitochondrial function, which indicates better microglial metabolic fitness.

### Microglia with PLCγ2 P522R variant have higher Ca^2+^ signalling and increased motility

**Fig. 6:**
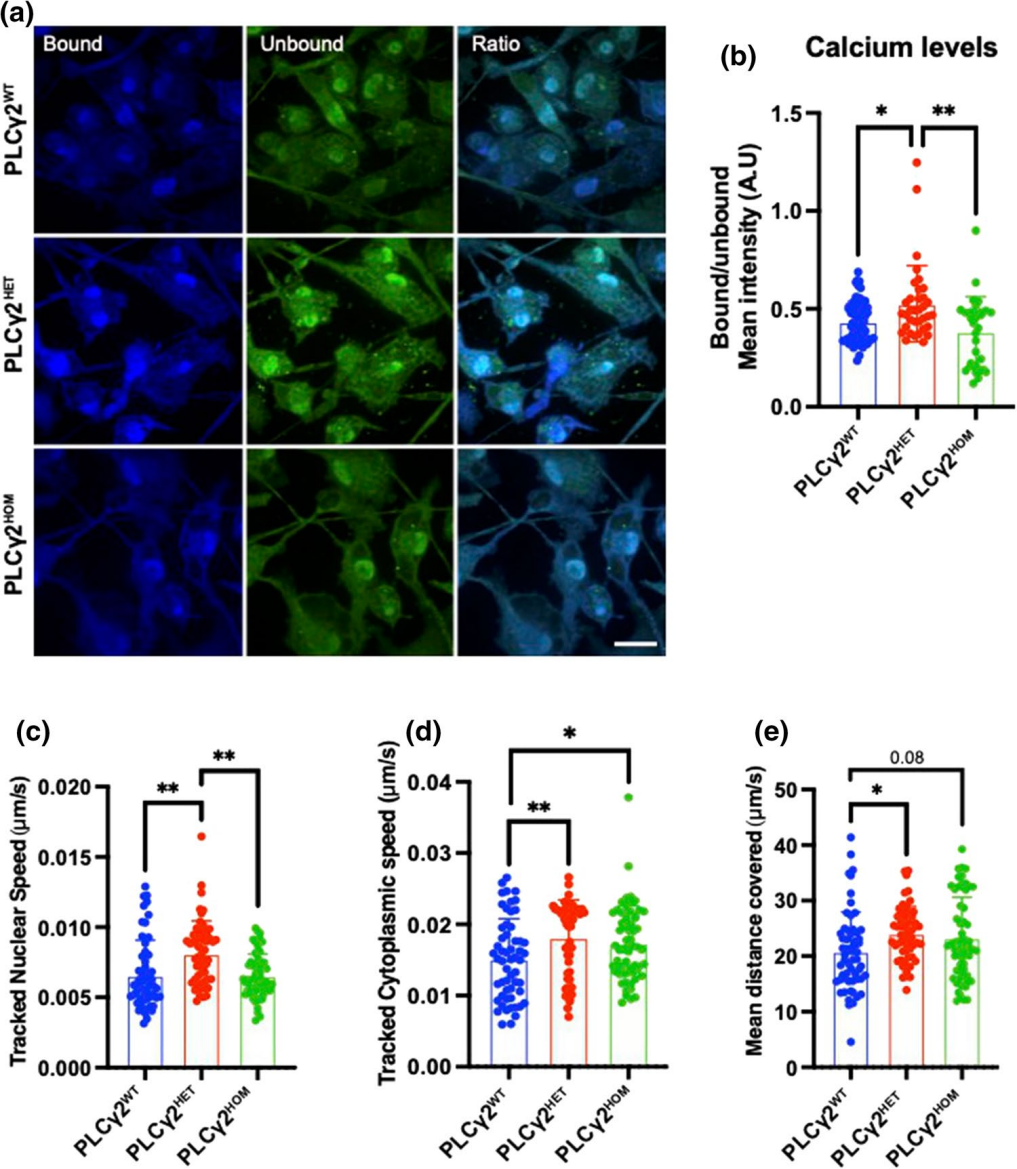
PLCγ2 ^P522R^ modulates basal Ca^2+^ levels and microglial motility. **a, b** Fura Red™ ratio-metric analysis of bound vs. unbound Ca^2+^ levels in PLCγ2^WT^, PLCγ2^HET^ and PLCγ2^HOM^ hiPSC-derived microglia. **c–e** Cellular motility assay assessing whole cell motility (nuclear tracking; **c**) and microglial ramification and surveillance (cytoplasmic tracking; **d**) as well as associated distance travelled (**e**) in PLCγ2^WT^, PLCγ2^HET^ and PLCγ2^HOM^ hiPSC-derived microglia. All data are presented as mean ± SD and were analysed using the Kruskal–Wallis with Dunns multiple comparisons test (*p < 0.05, **p < 0.01, *n* = 4–6, scale bar 50 μM)

PLCγ2 activity can induce calcium influx from the ER through the binding of IP3 to its receptor in the ER, IP3R [26]. This massive cytoplasmic Ca^2+^ influx is critical for various downstream signalling pathways that modulate numerous microglial functions. Hence, to investigate whether there are any differences in Ca^2+^ levels between the different PLCγ2 variants, we incubated the hiPSC-derived microglia with the cell permeable, light-excitable calcium sensor molecule Fura Red™. Live-cell imaging revealed significantly higher Ca^2+^ intensities, indicating higher Ca^2+^ levels, in PLCγ2^HET^ microglia compared to PLCγ2^WT^ cells, that was not seen in PLCγ2^HOM^ cells (Fig. 6A, B). Indeed, PLCγ2^HOM^ microglia had slightly reduced Ca^2+^ levels compared to PLCγ2^WT^ cells, although this did not reach significance (*p* = 0.1528). We also assessed changes in resting Ca^2+^ levels over time. Microglia were treated with Fura Red™ for 30 min and Ca^2+^ intensity monitored for 120 min. Consistent with our previous findings, both PLCγ2^HET^ and PLCγ2^WT^ microglia variants had persistently higher basal Ca^2+^ intensity compared to microglia with the PLCγ2^HOM^ variant (S. Figure 6).

The role of Ca^2+^ in cell migration and adhesion is well documented, hence given the differences in Ca^2+^ intensity among our PLCγ2 variants, we decided to investigate if this may impact on microglial motility. We assessed two parameters: nuclear tracking, for whole cell migration, and cytoplasmic tracking, for microglial ramification and surveillance. Microglia were tracked for 2 h and their motility assessed in untreated (homeostatic) conditions. Consistent with the changes observed in Ca^2+^ levels, PLCγ2^HET^ microglia had significantly higher nuclear speeds compared to PLCγ2^WT^ (*p* = 0.01) and PLCγ2^HOM^ (*p* = 0.007) variants, which themselves did not significantly differ (*p* = 0.99) (Fig. 6C). Cytoplasmic motility was also significantly enhanced in PLCγ2^HET^ cells, potentially indicative of increased surveillance activity (Fig. 6D).

In addition, by measuring total distance covered by the cells, we investigated whether the tracked speed had any connection to distance travelled. Consistent with our tracked speed findings, PLCγ2^HET^ significantly outperformed both PLCγ2^WT^ and PLCγ2^HOM^ variants in total distance covered (Fig. 6E). Our findings indicate that PLCγ2^HET^ microglia were more active in both surveillance and migration compared to PLCγ2^WT^ and PLCγ2^HOM^ microglia, and this could be tied to their housekeeping Ca^2+^ level.

## Discussion

In this study, we investigated the impact of the AD protective PLCγ2^P522R^ variant on human microglial cell functions. We used CRISPR editing to generate PLCγ2^HET^ and PLCγ2^HOM^ hiPSC cells, and differentiated them into microglia that exhibit functional and transcriptional profiles consistent with human microglia [21]. We were able to show that the presence of the *P522R* variant increased microglial uptake of the Aβ peptide, accompanied by a reduction in synapse pruning, two key pathological processes in AD. We provide evidence that this discrimination appears to be primarily driven by cargo size, and that cells expressing the PLCγ2^P522R^ variant have enhanced mitochondrial function and cell motility, potentially associated with increased intracellular Ca^2+^. We have also identified transcriptional changes in a number of genes associated with lipid metabolism, endosome/phagosome maturation and immune function in resting state PLCγ2^HET^ microglia, which highlight potential pathways that may underpin the protective capacity of the PLCγ2^P522R^ variant in AD. Crucially, we have shown that the impact of PLCγ2^P522R^ is dependent on gene dose, with heterozygous cells overall showing a more beneficial profile than homozygous cells.

The clearance of Aβ, along with other cellular debris, is an important feature of microglial phagocytic function in AD. Several other polymorphisms implicated as risk factors in AD, such as the R47H TREM2 mutation, result in alterations to microglial phagocytic capacity that lead to reduced clearance of Aβ aggregates and neuronal decline [27, 28]. In our hiPSC-derived microglia assay, PLCγ2^P522R^ enhanced the uptake of Aβ compared to PLCγ2^WT^ microglia, suggesting that the variant may positively impact directly on disease relevant pathology clearance, as previously suggested [15]. Consistent with this increased Aβ clearance, lysotracker levels, which indicate acidic vesicles such as lysosomes, were higher in both PLCγ2^HET^ and PLCγ2^HOM^ microglia variants compared to PLCγ2^WT^ cells. Interestingly, PLCγ2^HET^ microglia showed a more robust increase in lysotracker levels than PLCγ2^HOM^ cells, suggesting that there may be a narrow functional level (“sweet spot”) of PLCγ2^P522R^ that leads to improved outcomes. This is supported by the fact that while a number of the processes we assessed did appear to be ‘dose dependently’ modified by heterozygous vs. homozygous expression of the PLCγ2^P522R^ variant, other processes were preferentially impacted only in heterozygous cells. Indeed, a number of gain of function mutations in PLCγ2 have been linked to immune disorders, including PLAID (PLCγ2-associated antibody deficiency and immune dysregulation syndrome), APLAID (autoinflammation, antibody deficiency and immune dysregulation syndrome) and FCAS3 (familial cold autoinflammatory syndrome), as well as a subset of CVID (common variable immunodeficiency) patients [29–36]. These clearly indicate that a significant gain of function of PLCγ2 can lead to deleterious impacts on the immune system.

In the central nervous system, microglia shape neuronal synaptic connections and strength during development, through the removal of excess synapses [37]. While microglia-mediated synaptic pruning is a fundamental physiological process during development, its proposed reactivation during ageing has deleterious consequences and may account for much of the memory loss and cognitive decline observed in AD [24]. It has recently been reported that individuals with MCI (mild cognitive impairment), who carry the PLCγ2^P522R^ variant had better cognitive performance even in the presence of the APOE4 AD risk gene [38]. Here, we show hiPSC microglia with the PLCγ2^P522R^ variant had decreased synaptosome uptake compared to WT cells. Moreover, we were able to demonstrate that these PLCγ2^P522R^ microglia selectively spare synaptosomes while maintaining efficient clearance of Aβ when treated concurrently with both biological cargoes. These finding were replicated in BV2 cells over expressing exogenous human PLCγ2^WT^ or PLCγ2^P522R^, indicating a possible connection between increased expression of PLCγ2 and reduced synapse uptake. We provide further evidence of this in our neuronal and microglial co-culture study, where we saw a reduction in synaptic pruning by PLCγ2^P522R^ microglia, as evidenced by reduced levels of PSD95 within microglial cells. Interestingly, this was once again more robustly observed in PLCγ2^HET^ cells, supporting the notion of a ‘sweet spot’ for maximal beneficial impacts on microglial function.

The most prevailing question is how does the PLCγ2^P522R^ variant modulate diverse microglial functions to protect a degenerating brain? Our gene expression study highlights a number of different functional pathways that appear to be activated in PLCγ2^HET^ microglia, notably those relating to lipid metabolism, endosome/phagosome maturation and immune function. Proinflammatory environments and signalling are a prominent observation in AD brain pathology [39]. Our findings suggest a significant increase in the expression of the anti-inflammatory cytokine, interleukin-10 (IL-10), which is known to inhibit the synthesis of pro-inflammatory cytokines, and may promote the differentiation of microglia into the anti-inflammatory state, classically known as M2 or homeostatic state [40]. Interestingly, IL-10 deficiency has also been shown to exacerbate Tau pathology [41]. This, combined with our data, provides a potential route whereby the PLCγ2^P522R^ variant may impact at least in part on both primary pathologies associated with AD to reduce disease risk.

We also report significant increases in the expression of the chemokine receptor, CX3CR1 in PLCG2^HET^ cells. Within the CNS, this receptor is expressed exclusively by microglia, and binds to the neuronally expressed chemokine, CX3CL1 (fractalkine). The exact function of this interaction is not yet clear, but evidence suggests it plays an important role in the regulation of synaptic function, with CX3CR1 deficiency resulting in cognitive and LTP impairments indicative of altered synaptic plasticity [42, 43]. Certainly increased expression of CX3CR1 attenuates microglial inflammatory responses to LPS (liposaccharide) [44], while deletion results in an increased inflammatory response and phagocytic activity [45]. It is, therefore, possible that the increase in CX3CR1 we observe in resting state PLCγ2^HET^ microglia is linked to the reduction in synapse phagocytosis seen in these cells, although more work is required to unpick the mechanism that may underpin this.

A growing body of evidence suggests that lipid metabolism is crucial to fuel microglial functions such as phagocytosis, and can vary depending upon the activation status of the cell (reviewed in [46]). Previous studies have shown that PLCG2 acts downstream of TREM2, and knock out of either protein results in lipid accumulation deficits and a failure to activate key lipid processing genes such as LIPA and APOC1 [14], as well as a generalised alteration in the overall lipidome of the cells. In the current study, heterozygous PLCγ2^P522R^ induced increases in the expression of genes associated with phagocyte maturation (RAB5, RAB7) and various aspects of lipid metabolism (LIPA, APOE, PLIN2) in resting state microglia, an effect which is not seen in homozygous cells. This may suggest that these heterozygous cells are ‘primed’ to more readily respond to an inflammatory challenge, and can, therefore, more efficiently uptake and phagocytose targets such as Aβ when required, a hypothesis supported by recent work suggesting that PLCγ2^P522R^ may promote microglial activation [17].

It is important to note that significant changes in gene expression in our cells were primarily observed when comparing PLCG2^HET^ and PLCG2^WT^ cells, and under our current experimental conditions, we cannot rule out an impact of CRISPR editing per se on this expression profile. However, while no significant changes were observed between PLCG2^HET^ and PLCG2^HOM^ cells, Il-10, CX3CR1 and RAB5 showed trends towards enhanced expression in PLCG2^HET^ cells. Our cell assays show that heterozygous expression of PLCG2^P522R^ is the most beneficial in AD-like phenotypes, suggesting that expression changes in these three genes may play a key role in modulating microglial cell function to help protect against LOAD.

It is also important to acknowledge that the gene expression changes we observed in the PLCγ2^HET^ microglia were all seen in resting state cells, without LPS or other disease relevant immune challenge. These expression changes are likely to be important factors in the protective capacity of PLCγ2^P522R^. LOAD develops over many years, rather than arising as a result of a single acute challenge, thus these low level ongoing changes likely reflect the chronic impact of the variant on general microglial function. However, it is probable that additional changes will be observed in response to immune challenge, and further work is required to elucidate these changes, and understand how they impact on microglial function, to improve target cargo uptake and preserve synapses. Indeed, we identified a number of additional genes that trended towards a difference between PLCγ2^HET^ and PLCγ2^WT^ cells, that may indicate a subset of genes ‘primed’ to respond to immune challenge. Loss of PLCG2 results in a spectrum of gene expression changes in microglia both at rest, and in response to TLR stimulation [14], and intriguingly, this TLR induced profile appears distinctly different from that seen with TREM2 knockout, suggesting that PLCG2 may interact independently with both TREM2 and TLR linked pathways, although we did not see significant changes in the basal expression of TREM2 or TLR4, or functionally associated proteins such as NLRP3 in our cells. A broad spectrum of gene expression changes have also been reported in homozygous PLCγ2^P522R^ knock-in mice [17]. However, given our findings, which suggest that heterozygosity is important for maximal benefits from the variant, examining the difference between heterozygous and homozygous PLCγ2^P522R^ basal and stimulated gene expression profiles may be invaluable in more clearly identifying specific functional changes responsible for the protective impact of PLCγ2^P522R^ in LOAD.

The highly dynamic nature of microglia makes them heavily dependent on efficient energy expenditure to meet basic housekeeping demands. To maintain brain homeostasis and surveillance, microglia require energy-demanding cytoskeleton remodelling, necessary for extending and retracting their ramified processes, to constantly scan their surroundings [47, 48]. While in a resting state, microglia use mitochondrial respiration, specifically OXPHOS (oxidative phosphorylation) as their main source of energy, although resting state is a misconception, since microglia are never quiescent, rather they are constantly engaged in parenchymal surveillance [48]. Our findings indicate that microglia expressing PLCγ2^P522R^ in general show enhanced mitochondrial respiration as well as significantly higher ATP production, which was not associated with increases in mitochondrial number but rather changes in mitochondrial function.

Mitochondrial function directly correlates with cytoplasmic Ca^2+^ levels, which could underlie the enhancement we observed in the PLCγ2^P522R^ cells. PLCγ2 is a direct modulator of Ca^2+^ influx from the endoplasmic reticulum (ER) via its hydrolysis of PIP2, leading to the production of IP3, which then interacts with its receptor IP3R1 [49]. Recent reports have demonstrated that the presence of the PLCγ2^P522R^ variant in microglia enhances Ca^2+^ release in response to physiological stimuli [15]. In our study, we found that PLCγ2^HET^ microglia showed higher levels of basal intracellular Ca^2+^ than PLCγ2^WT^ cells in the absence of any stimulus, supporting the notion that the PLCγ2^HET^ cells may be ‘primed’ to respond more rapidly to an inflammatory challenge. This increase was not observed in the PLCγ2^HOM^ cells, once again highlighting the delicate balance of P522R dose on PLCγ2 function. Interestingly, PLCγ2^HOM^ cells did demonstrate a greater enhancement of mitochondrial function than the PLCγ2^HET^ cells, in the absence of any change in Ca^2+^ levels. This separation between Ca^2+^ levels and mitochondrial performance suggests that other as yet unidentified factors may play a key role in modulating microglial cell energy homeostasis.

Ca^2+^ currents have also been implicated in the regulation of microglial motility (reviewed in [50]). Consistent with the increase, we see in intracellular Ca^2+^ in PLCγ2^HET^ cells, they also demonstrated enhanced mobility compared to PLCγ2^WT^ cells. Both whole cell movement, detected via nuclear tracking, as well as surveillance, detected via assessing cytoplasmic movement alone, were enhanced. PLCγ2^HOM^ cells also showed increased surveillance, but their nuclear movement did not differ from PLCγ2^WT^ cells, once again highlighting the delicate functional balance between heterozygous and homozygous expression of the P522R variant.

Our study is the first to suggest that the protective nature of the PLCγ2^P522R^ variant in AD may reflect a selective increase in microglial clearance of Aβ, coupled with a preservation of synapses. This is accompanied by an increase in mitochondrial fitness and microglial motility, suggesting that PLCγ2^P522R^ may enhance basal microglial functioning, leading to a relative protection against the accumulation of pathogenic proteins seen in both healthy ageing and AD. A number of resting state transcriptional changes are associated with this altered function, which appears to be critically dependent on PLCγ2^P522R^ ‘dose’, with heterozygous expression of the variant optimal.

## Conclusions

These data highlight the complex role of microglia in health and disease, and the importance of understanding how risk factors such as PLCγ2^P522R^ exert their effects, and how protein or variant dose can play a key role in modifying cellular functions. It suggests that more investigation is needed into the delicate balance of PLCG2 and microglial function in health and disease, and shows the need for caution and rigorous testing when targeting these pathways for therapeutic development.

## Supplementary Information

Below is the link to the electronic supplementary material.

Supplementary file1 (DOCX 9144 KB)

## Data Availability

All data generated or analysed during this study are included in this published article [and its supplementary information files].

## Abbreviations

Aβ: Amyloid beta
ABCA1: ATP binding cassette subfamily A member 1
ABCA7: ATP binding cassette subfamily A member 7
ABI3: ABI family member 3
AD: Alzheimer’s disease
AHR: Aryl-hydrocarbon receptor
AHRR: Aryl-hydrocarbon receptor repressor
AiF1: Allograft inflammatory factor 1
APLAID: Autoinflammation, antibody deficiency and immune dysregulation syndrome
APOE: Apolipoprotein E
ARNT: Aryl-hydrocarbon receptor nuclear translocator
ATP: Adenosine 5″ triphosphate
B2M: Beta-2-microglobulin
C1QA: Complement C1q subcomponent subunit A
CD14: Cluster of differentiation 14
CD33: Cluster of differentiation 33
CD52: Cluster of differentiation 52
CD68: Cluster of differentiation 68
CD9: Cluster of differentiation 9
CLCN7: Chloride voltage-gated channel 7
CNS: Central nervous system
CSF1: Colony-stimulating factor 1
CST3: Cystatin 3
CTSB: Cathepsin B
CTSD: Cathepsin D
CVID: Common variable immunodeficiency
CX3CR1: C-X3-C motif chemokine receptor 1
CYP1A1: Cytochrome P450 family 1 subfamily a member 1
CYP1B1: Cytochrome P450 family 1 subfamily b member 1
DAM: Disease-associated microglia
ER: Endoplasmic reticulum
FABP5: Fatty acid-binding protein 5
FCAS3: Familial cold autoinflammatory syndrome
HEXB: Hexosaminidase subunit beta
hiPSC: Human induced pluripotent stem cell
HK3: Hexokinase 3
IBA1: Ionised calcium-binding adaptor molecule 1
IFNG: Interferon gamma
IL-10: Interleukin-10
IL1B: Interleukin 1 beta
IL4: Interleukin 4
IL6: Interleukin 6
IP3R: Inositol trisphosphate receptor
LAMP1: Lysosomal-associated membrane protein 1
LAMP2: Lysosomal-associated membrane protein 1
LIPA: Lipase A, lysosomal acid type
LOAD: Late-onset Alzheimer’s disease
MCI: Mild cognitive impairment
NFKB: Nuclear factor kappa B subunit
NLRP3: NLR family pyrin domain containing 3
OCR: Oxygen consumption rate
P2RY12: Purinergic receptor P2Y12
PFKFB1: 6-Phosphofructo-2-kinase/fructose-2,6-biphosphatase 1
PFKFB3: 6-Phosphofructo-2-kinase/fructose-2,6-biphosphatase 3
PLAID: PLCγ2-associated antibody deficiency and immune dysregulation syndrome
PLCG2/PLCγ2: Phospholipase C gamma 2
PLIN2: Perilipin 2
PSD95: Postsynaptic density protein 95
RAB5: Member RAS oncogene family5
RAB7: Member RAS oncogene family7
SIRPa: Signal regulatory protein α
SPI1: Spi-1 proto-oncogene
TFB2M: Transcription factor B2, mitochondrial
TLR4: Toll-like receptor 4
TMEM119: Transmembrane protein 119
TOMM20: Translocase of outer mitochondrial membrane 20
TREM2: Triggering receptor expressed on myeloid cells 2
TSPO: Translocator protein
TYROBP: Protein tyrosine kinase-binding protein
WT: Wild type
