# Supplementary material for: Heterozygous expression of the Alzheimer’s disease-protective PLCγ2 P522R variant enhances Aβ clearance while preserving synapses
Source: Cell Mol Life Sci. 2022 Jul 27;79(8):453. doi: 10.1007/s00018-022-04473-1 (PMC9329165; doi:10.1007/s00018-022-04473-1)
Supplement: Supplementary file 1 — Supplementary file1 (DOCX 9144 KB) [file 18_2022_4473_MOESM1_ESM.docx]

Supplementary Methods.

Plasmids

The pUCM-AAVS1-TO-hNGN2 construct, which was used to generate cortical neurons, was a gift from Michael Ward (Addgene plasmid #105840; http://n2t.net/addgene:105840 ; RRID:Addgene_105840). The pRR-EF1a-Puro is based on the pRR-Puro plasmid (Flemr and Buhler, 2015) and was ordered by gene synthesis (General Biosystems). The pRR-EF1a-Puro-PLCγ2_P522R plasmid was generated by annealing two oligonucleotides (supplementary Table2) containing the CRISPR target site followed by cloning in SalI and SpeI digested pRR-EF1a-Puro. The PLCγ2-P522R exchange matrix (template) was custom designed and synthesised (GeneArt, ThermoFisher), containing homology arms of 650bp upstream and downstream of the P522R conversion site. The PLCγ2^WT^ overexpression construct pCMV6-PLCG2-Myc-DDK was purchased from Origene (RC200442). The PLCγ2^P522R^ overexpression construct was generated using pCMV6-PLCG2-Myc-DDK, mutagenesis primers (supplementary Table2) and the Q5 site directed mutagenesis kit (E0554S, NEB), followed by transformation in DH5α chemically competent cells (ThermoFisher). The PLCγ2 control cDNA was designed with a frameshift mutation that disrupts the open reading frame followed by gene synthesis (TwistBioscience).

*Flemr, M., and Buhler, M. (2015). Single-Step Generation of Conditional Knockout Mouse Embryonic Stem Cells. Cell Rep 12, 709-716. 10.1016/j.celrep.2015.06.051.*

hiPSC Karyotyping

hiPSC lines (PLCγ2^WT^, PLCγ2^HET^ and PLCγ2^HOM^) were analysed to detect any major karyotypic defect reported in human iPS cells using a hiPSC karyotyping kit (#07550, Stemcell Technologies) according to the manufacturers protocol. In brief genomic DNA was harvested from the three iPS cell lines (PLCγ2^WT^, PLCγ2^HET^ and PLCγ2^HOM^) was harvested using DNeasy Blood & Tissue (69506, QIAGEN) and used immediately. 12μL of ROX reference dye was added to the qPCR Master Mix and briefly vortexed for 5min and placed on ice (protected from light). In a microcentrifuge tube 300ng of genomic DNA from the samples and a control was prepared and adjusted the volume to 90μL using nuclease free water. 150μL of Master Mix and Dye was added to each sample and control and mixed by pipetting up and down. Primer-probes were prepared by briefly centrifuging at 750 x g for 30sec and 33μL of TE Buffer was added to each tube. The solution was then mixed by pipetting up and down to ensure complete suspension of the primer-probes. After calculating the number of reactions required, for each sample and control with three technical replicates, in a new microcentrifuge working primer-probe solution was prepared by adding the necessary volume of nuclease free water and primer-probe. In a 384 well qPCR plate, 8μL of the prepared Master-Mix-Dye, for each sample and control, was added to the bottom edge of each well. Working with one primer at a time, 2μL of the prepared primer-probes were added to each appropriate well and the plate was covered with optical adhesive film and centrifuged at 1000 x g for 1min. The plate was run on a standard run of 95^o^C polymerisation activation followed by 40 cycles of denaturing at 95oC and annealing/extension at 60^o^C. Results were analysed on Stemcell technologies web analysis platform (https://shiny.stemcell.com/ShinyApps/psc_genetic_analysis_app) and results are presented as a fold-change compared to a standardised female control (Stemcell Technologies).

Supplementary Table 1: List of sequencing and cloning primers

| **GENE** | **FWD** | **REV** |
| --- | --- | --- |
| PLCG2 (Outside HA-sequencing primers) | AAAGGGGGATGTGCTGCAAGGC | GCTGTTTCCTTGCGTATTGGGCG |
| PLCG2 (Inside HA – sequencing primers) | CCTCATGGCTCTCAGACTTC | CTAGAAGAGCTAGACAAATGAAGC |
| PLCG2 (Over-expression plasmid – sequencing primers) | CGACGTCGTGCAGGCCATCAAA | TAGGAGTCGCTCCCCTCTCGCT |
| PLCG2-P522R (Mutagenesis Primers) | CAGGATATAC**g**CCCTACAGAACTAC | GGGCACTTCCTCCTCCAT |
| PRR-Puro-PLCG2 primers | TCGACCCCCTACAGAACTACATT  TTGGGGATGCAAAGCGTGATGA | CTAGTCATCACGCTTTGCATCCC  CAAAATGTAGTTCTGTAGGGGG |
| (pRR-EF1a-Puro-[R495X]) |  |  |

Supplementary Table 2: List of primary and secondary antibodies

| **Antibody** | **Host** | **Reference number** | **Manufacturer** | **Dilution** | |
| --- | --- | --- | --- | --- | --- |
| Anti PLCG2 | Mouse | sc-5283 | Santacruz | WB 1:1000 | ICC 1:200 |
| Anti TOMM20 | Rabbit | ab186735 | Abcam | WB 1:1000 |  |
| Anti-Iba1 | Rabbit | 019-19741 | Waco |  | ICC 1:500 |
| Anti TMEM119 | Rabbit | ab185333 | Abcam |  | ICC 1:200 |
| MAP2 | Chicken | ab92434 | Abcam |  | ICC 1:2000 |
| PSD95 | Mouse | MA1-045 | ThermoFisher |  | ICC 1:200 |
| Hoechst |  | 62249 | ThermoFisher |  | ICC 1:12000 |
| Anti B-Actin | Mouse | ab8226 | Abcam | WB 1:10000 |  |
| Anti B-Actin | Rabbit | ab8227 | Abcam | WB 1:10000 |  |
| Anti-Mouse-680 | Goat | 926-68070 | LICOR | WB 1:10000 |  |
| Anti-Rabbit-680 | Goat | 926-68071 | LICOR | WB 1:10000 |  |
| Anti-Mouse-800 | Goat | 926-32210 | LICOR | WB 1:10000 |  |
| Anti-Rabbit-800 | Goat | 926-32211 | LICOR | WB 1:10000 |  |
| Anti-Mouse-647 | Goat | A-21235 | ThermoFisher |  | ICC 1:500 |
| Anti-Rabbit-647 | Goat | A-27040 | ThermoFisher |  | ICC 1:500 |
| Anti-Mouse-488 | Goat | A-11001 | ThermoFisher |  | ICC 1:500 |
| Anti-Rabbit-488 | Goat | A-11008 | ThermoFisher |  | ICC 1:500 |
| Anti-Mouse-568 | Goat | A-11004 | ThermoFisher |  | ICC 1:500 |
| Anti-Rabbit-568 | Goat | A-11011 | ThermoFisher |  | ICC 1:500 |
| Anti-Chicken-568 | Goat | A-32932 | ThermoFisher |  | ICC 1:500 |

Supplementary Table 3: List of RT-qPCR Primers

| **GENE** | **FWD** | **REV** |
| --- | --- | --- |
| PLCG2 | CATCCTATATGGCACTCAGTTCG | TCCTGGTGTAAGATTTTCAAGCC |
| Plcg2 | GTGGACACCCTTCCAGAATATG | ACCTGCCGAGTCTCCATGAT |
| HEXB | GTCAGAGTGTGATGCTTTCCC | TAAACCTCGTAATGCTCCCCA |
| HK3 | GGACAGGAGCACCCTCATTTC | CCTCCGAATGGCATCTCTCAG |
| PFKFB3 | AGCCCGGATTACAAAGACTGC | GGTAGCTGGCTTCATAGCAAC |
| PFKFB1 | TACCAGAGAACGACGGTCACT | CTGCAATTATGCCAGGGTCATTA |
| TFB2M | GACCACTTACGTTCATTGACTCC | CAGGGTTTCATCATACAGCCAT |
| TSPO | TCTACTCAGCCATGGGGTAC | CAGCTGCCCAGTGTAGAGG |
| ABCA1 | AGCTACCCACCCTATGAACAAC | ACAGGGGTTGTTGGCATTAC |
| ABCA7 | GCTGCTGCTCTGGAAGAATTTC | AAGTGGCATTCATGGTGCTC |
| LIPA | TCTGGACCCTGCATTCTGAG | CACTAGGGAATCCCCAGTAAGAG |
| APOE | GTTGCTGGTCACATTCCTGG | GCAGGTAATCCCAAAAGCGAC |
| FABP5 | TGAAGGAGCTAGGAGTGGGAA | TGCACCATCTGTAAAGTTGCAG |
| PLIN2 | TTGCAGTTGCCAATACCTATGC | CCAGTCACAGTAGTCGTCACA |
| CTSB | CCCAGAGAGTTATGTTTACCG | TGGTCTCTGATCTCTTTGATG |
| CTSD | AGGGCGAGTACATGATCC | GGACAGCTTGTAGCCTTTG |
| LAMP1 | AGCTCCAAAGAAATCAAGAC | GGGTGCCACTAACACATC |
| LAMP2 | TGGTGTTGCAGCTGTTGTTG | CGCACAGCTCCCAGGACTA |
| CCLN7 | AAGGGCAATATCGACAAG | TCTATGAAAGCCACAATCAC |
| RAB7 | TGGTGTCCACGTTCTACAAGG | CCAGGGCTTCAAAAGACTCCA |
| RAB5 | CAAGGCCGACCTAGCAAATAA | GATGTTTTAGCGGATGTCTCCAT |
| CD68 | CTTCTCTCATTCCCCTATGGACA | GAAGGACACATTGTACTCCACC |
| TLR4 | AGTTGATCTACCAAGCCTTGAGT | GCTGGTTGTCCCAAAATCACTTT |
| CX3CR1 | AGTGTCACCGACATTTACCTCC | AAGGCGGTAGTGAATTTGCAC |
| P2RY12 | TTTGTGTGTCAAGTTACCTCCG | CTGGTGGTCTTCTGGTAGCG |
| NLRP3 | CCACAAGATCGTGAGAAAACCC | CGGTCCTATGTGCTCGTCA |
| CD52 | TCTTCCTCCTACTCACCATCAG | CCTCCGCTTATGTTGCTGGA |
| CD9 | TCCACTATGCGTTGAACTGCT | GGTTTCGAGTACGTCCTTCTTG |
| TREM2 | GGTCAGCACGCACAACTTG | CGCAGCGTAATGGTGAGAGT |
| TYROBP | ACTGAGACCGAGTCGCCTTAT | ATACGGCCTCTGTGTGTTGAG |
| B2M | GAGGCTATCCAGCGTACTCCA | CGGCAGGCATACTCATCTTTT |
| CD33 | GGCCACTCCAAAAACCTGAC | GACAACCAGGAGAAGATCGGG |
| C1QA | TCTGCACTGTACCCGGCTA | CCCTGGTAAATGTGACCCTTTT |
| IL1B | TTCGACACATGGGATAACGAGG | TTTTTGCTGTGAGTCCCGGAG |
| IFNγ | TCTTTGGGTCAGAGTTAAAGCCA | TTCCATCTCGGCATACAGCAA |
| IL4 | CCGTAACAGACATCTTTGCTGCC | GAGTGTCCTTCTCATGGTGGCT |
| IL6 | CCTGAACCTTCCAAAGATGGC | TTCACCAGGCAAGTCTCCTCA |
| IL10 | TCAAGGCGCATGTGAACTCC | GATGTCAAACTCACTCATGGCT |
| NF-KB | GTCAAAAACGCCACCTCTCAA | CTCGCATGGAATTTGGAACCG |
| AHR | ACATCACCTACGCCAGTCG | CGCTTGGAAGGATTTGACTTGA |
| ARNT | CTGCCAACCCCGAAATGACAT | CGCCGCTTAATAGCCCTCTG |
| AHRR | GCGCCTCAGTGTCAGTTACC | GAAGCCCAGATAGTCCACGAT |
| CYP1A1 | GGTCAGCATGTGCCCAATCA | TCGGCCACGGAGTTTCTTC |
| CYP1B1 | AAGTTCTTGAGGCACTGCGAA | GGCCGGTACGTTCTCCAAAT |
| SIRPa | GGCCTCAACCGTTACAGAGAA | GTTCCGTTCATTAGATCCAGTGT |
| CST3 | GTCGGCGAGTACAACAAAGC | CACCCCAGCTACGATCTGC |
| AIF1 | ATGAGCCAAACCAGGGATTTAC | GGGATCGTCTAGGAATTGCTTGT |
| CD14 | GACCTAAAGATAACCGGCACC | GCAATGCTCAGTACCTTGAGG |
| SPI1 | GTGCCCTATGACACGGATCTA | AGTCCCAGTAATGGTCGCTAT |
| TMEM119 | CGGCCTATTACCCATCGTCC | CTGGGCTAACAAGAGAGACCC |
| CSF1 | TGGCGAGCAGGAGTATCAC | AGGTCTCCATCTGACTGTCAAT |

Supplementary Figures


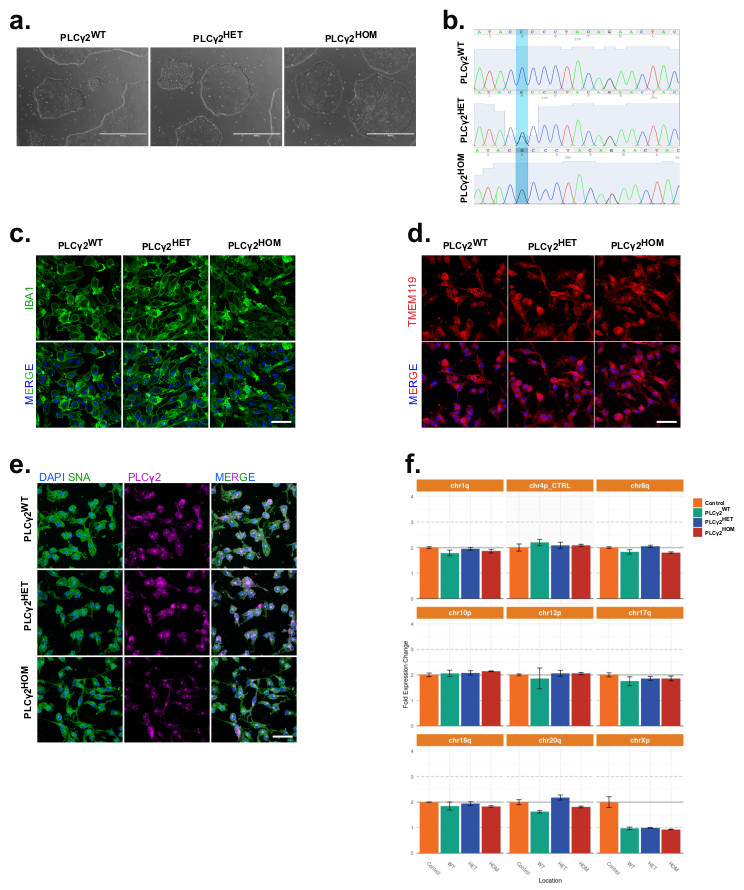


**SFig 1: Validation of PLCγ2 ^P522R^ modified iPSC derived microglia**

**a,** Representative brightfield image of healthy Bioni-10 iPSCs displaying characteristic robust colony formation. **b,** Representative sequencing trace of Bioni-10 WT, P522R heterozygote, and P522R homozygote iPSC line, highlighting a Proline (P) to Arginine (R) conversion after CRISPR editing. **c-e,** Representative confocal images of differentiated microglia expressing key microglia markers IBA1 (c) and TMEM119 (d), as well as PLCγ2 (e). qPCR karyotyping analysis of common karyotypic abnormalities in iPSC cells demonstrating no differences between Bioni-10 WT, P522R heterozygote, and P522R homozygote iPSC lines compared to the commercially provided control, apart from that conferred by cell sex (crhXp) (f).

**SFig.2:** Overexpression of PLCγ2 result in increased Aβ uptake and reduced synaptosome uptake in BV2 cells.

**a-c,** Western blot (a-b) and RT-qPCR (c) analysis of PLCγ2 expression levels in BV2 cells transfected with a control, PLCγ2^WT^ or PLCγ2^P522R^ expression construct. Expression levels were normalised to β-actin. Data is presented as mean ± SD. Western blot data was analysed using one-way ANOVA with the Tukey multiple comparisons test, and RT-qPCR data was analysed using the Kruskal-Wallis test with Dunns multiple comparisons. **d-e,** Fluorescent confocal analysis of Aβ_1-42_ HyLite Fluor 647 uptake (magenta) in BV2 cells following transfection with a PLCγ2^WT^ or PLCγ2^P522R^ expression construct. Internalised Aβ is expressed as a proportion of total cell area detected using CellBrite^®^ (green) (Scale bar 50μM). **f-g,** Fluorescent confocal analysis of tdTomato-synaptosome uptake (red) in BV2 cells following transfection with a PLCγ2^WT^ or PLCγ2^P522R^ expression construct. Internalised synaptosome levels are expressed as a proportion of total cell area, detected using CellBrite^®^ (green) (Scale bar 50μM). **h,** Representative high power confocal images of BV2 cells transfected with a control, PLCγ2^WT^ or PLCγ2^P522R^ expression construct and incubated with Aβ_1-42-_647 and synaptosomes, showing synaptosomes mainly localized to the membrane (arrows) while Aβ is fully internalised (arrowheads) (scale bar 20μm). Data is presented as mean ± SD and unless otherwise described was analysed using the Kruskal-Wallis with Dunns multiple comparisons test. (*p<0.05, **p<0.01, n=3-9).

**SFig.3:** PLCγ2 overexpression in BV2 cells affects cells size.

**a-b,** Confocal imaging of BV2 cells following transfection with a control, PLCγ2^WT^ or PLCγ2^P522R^ expression construct. Total cell area was visualised and quantified using CellBrite^®^ (green). Data is presented as mean ± SD and was analysed using the Kruskal-Wallis with Dunns multiple comparisons test. (*p<0.05, **p<0.01, n=6, Scale bar 50μm).

**SFig.4:** PLCγ2 overexpression size-dependently modulates uptake of non-biological cargoes.

**a-d,** Fluorescent confocal analysis of 4kDa (a-b) or 150kDa (c-d) FITC-dextran (green) uptake by BV2 cells following transfection with a control, PLCγ2^WT^ or PLCγ2^P522R^ expression construct. Internalised dextran is expressed as a proportion of total cell area, detected using Wheat Germ Agglutinin (magenta). **e-f,** Fluorescent confocal analysis of Alexa Fluor^TM^ 594-zymosan A Bioparticles^TM^ (red) uptake by BV2 cells following transfection with a PLCγ2^WT^ or PLCγ2^P522R^ expression construct. Internalised zymosan particles are expressed as a proportion of total cell area, detected using CellBrite^®^ (green). All data is presented as mean ± SD and was analysed using the Kruskal-Wallis with Dunns multiple comparisons test. (*p<0.05, **p<0.01, n=3, Scale bar 50μm).

**SFig.5:** PLCγ2 modulates mitochondrial function in BV2 cells.

**a-e,** Real time oxygen consumption rate (OCR) at baseline and in response to oligomycin (ATP-synthase inhibitor), FCCP (mitochondrial membrane uncoupler) and rotenone/antimycin-A (Complex I and III inhibitors) in BV2 cells following transfection with a control, PLCγ2^WT^ or PLCγ2^P522R^ expression construct. Calculated basal respiration (b), maximal respiration (c) ATP production (d) and proton leak (e) are reported. Data is presented as mean ± SD and was analysed using the Kruskal-Wallis with Dunns multiple comparisons test. (*p<0.05, **p<0.01, n=3-4).

**SFig.6:** PLCγ2 modulates Ca^2+^ level in iPSC derived microglia.

**a,** Longitudinal assessment of Fura Red^TM^ ratiometric analysis of bound vs. unbound Ca^2+^ levels in PLCγ2^WT^, PLCγ2^HET^ and PLCγ2^HOM^ hiPSC derived microglia, monitored every 10 minutes for 120 minutes. **b,** Mean Ca^2+^ intensity across the whole 2h period. All data is presented as mean ± SD and was analysed using the Kruskal-Wallis with Dunns multiple comparisons test. (*p<0.05, **p<0.01, n=3).
